# The role of nano-perovskite in the negligible thorium release in seawater from Greek bauxite residue (red mud)

**DOI:** 10.1038/srep21737

**Published:** 2016-02-22

**Authors:** Platon N. Gamaletsos, Athanasios Godelitsas, Takeshi Kasama, Alexei Kuzmin, Markus Lagos, Theo J. Mertzimekis, Jörg Göttlicher, Ralph Steininger, Stelios Xanthos, Yiannis Pontikes, George N. Angelopoulos, Charalampos Zarkadas, Aleksandr Komelkov, Evangelos Tzamos, Anestis Filippidis

**Affiliations:** 1 Center for Electron Nanoscopy, Technical University of Denmark, 2800 Kongens Lyngby, Denmark; 2 Faculty of Geology & Geoenvironment, National and Kapodistrian University of Athens, Zografou Campus, 15784 Athens, Greece; 3 Institute of Solid State Physics, University of Latvia, Kengaraga str. 8, 1063 Riga, Latvia; 4 Karlsruhe Institute of Technology, Institute for Nuclear Waste Disposal, Hermann-von-Helmholtz-Platz 1, 76344 Eggenstein-Leopoldshafen, Germany; 5 Faculty of Physics, National and Kapodistrian University of Athens, Zografou Campus, 15784 Athens, Greece; 6 Karlsruhe Institute of Technology, ANKA Synchrotron Radiation Facility, Hermann-von-Helmholtz-Platz 1, 76344 Eggenstein-Leopoldshafen, Germany; 7 Department of Electrical and Computer Engineering, Nuclear Technology Laboratory, Aristotle University of Thessaloniki, 54124 Thessaloniki, Greece; 8 Department of Automation Engineering, Alexander Technological Educational Institute of Thessaloniki, 57400 Thessaloniki, Greece; 9 KU Leuven, Department of Materials Engineering, Kasteelpark Arenberg 44, 3001 Leuven, Belgium; 10 University of Patras, Department of Chemical Engineering, 26500 Rio, Greece; 11 PANalytical B.V., 7600 AA Almelo, The Netherlands; 12 School of Geology, Aristotle University of Thessaloniki, 54124, Thessaloniki, Greece

## Abstract

We present new data about the chemical and structural characteristics of bauxite residue (BR) from Greek Al industry, using a combination of microscopic, analytical, and spectroscopic techniques. SEM-EDS indicated a homogeneous dominant “Al-Fe-Ca-Ti-Si-Na-Cr matrix”, appearing at the microscale. The bulk chemical analyses showed considerable levels of Th (111 μg g^−1^), along with minor U (15 μg g^−1^), which are responsible for radioactivity (355 and 133 Bq kg^−1^ for ^232^Th and ^238^U, respectively) with a total dose rate of 295 nGy h^−1^. Leaching experiments, in conjunction with SF-ICP-MS, using Mediterranean seawater from Greece, indicated significant release of V, depending on S/L ratio, and negligible release of Th at least after 12 months leaching. STEM-EDS/EELS & HR-STEM-HAADF study of the leached BR at the nanoscale revealed that the significant immobility of Th^4+^ is due to its incorporation into an insoluble perovskite-type phase with major composition of Ca_0.8_Na_0.2_TiO_3_ and crystallites observed in nanoscale. The Th *L*_III_-edge EXAFS spectra demonstrated that Th^4+^ ions, which are hosted in this novel nano-perovskite of BR, occupy Ca^2+^ sites, rather than Ti^4+^ sites. That is most likely the reason of no Th release in Mediterranean seawater.

Worldwide, the refining of bauxite ore deposits to alumina through the Bayer process results in production of huge quantities of a solid metallurgical bauxite residue (BR), the so-called “red mud”. It is estimated that 2 metric tones of BR are produced per ton of alumina (Al_2_O_3_) and, moreover, that up to 120 × 10^6^ metric tones of this residual material are deposited every year by the global Al industry e.g.^1^,^2^. The sustainable storage of BR in enormous quantities raises severe concerns on the design, construction and operation of their reservoir, directly related to economic, spatial, technological and environmental issues e.g.^3^,^4^. For instance, the major deadly accident happened in Hungary around the Ajkai Timföldgyár alumina plant on 4^th^ October 2010 e.g.^5^,^6^. In that accident, it was proved that BR acted as a significant source of V, and As in the environment^1^,^7^ whereas radioactivity issues, due to ^232^Th, ^238^U, and ^40^K, have also raised concern^8^.

Nowadays, according to U.S. Geological Survey (USGS), Greece is considered the 16^th^ alumina (Al_2_O_3_) producer in the world and the 4^th^ among the E.U. member-states. In Greece, the production of BR from the “Aluminium of Greece S.A.” at its processing plant in Agios Nikolaos area (Antikyra bay, Gulf of Corinth) is approx. 500–680 × 10^3^ metric tones per year^9^,^10^. As per previous regulations, the plant was allowed to pump the BR directly to the bottom of the sea, at the depth of >100 m. Since 2007, this practice has gradually ceased and through substantial investments (i.e., four filter presses to deliver a semi-dry cake, a new disposal area, a number of research projects for valorization trajectories and more) BR is currently industrially utilized in cement production, with ongoing efforts in the area of ceramics and metallurgy e.g.^11^,^12^,^13^. Due to the former disposal practice, millions of metric tones of BR are still remaining on the sea bed of the Antikyra bay e.g.^14^,^15^, fostering the debate on the potential impact of BR to the marine environment. At the same time, the BR potentially acts as alternative resource of strategic metals and that is of great concern, too. Previous studies have been carried out to elucidate the basic chemical composition and distribution of the discharged BR onto the bottom of sea e.g.^14^,^15^ and to address the issue of the nature of actinides in BR^11^,^12^. Claims on elevated radioactivity, mainly due to ^232^Th-series, and potential release of radionuclides in the environment of the Antikyra Bay were also indicated^16^,^17^,^18^. It is noteworthy to mention that an industrial scale pilot plant (the so-called “ENEXAL” BR treatment process e.g.^19^,^20^), resulting in the full conversion of BR into pig iron and mineral wool, has been recently applied mainly by the “Aluminium of Greece S.A.”. On the other hand, the determination of rare earth elements/REEs (i.e., lanthanides + Y + Sc) in BR, and the subsequent recovery of REEs using acids has also been reported^3^,^4^,^21^,^22^,^23^,^24^,^25^,^26^.

The fact that Th, which is primarily present in the minerals of the initial bauxite ore e.g.^27^,^28^, is subsequently transferred into BR through the Bayer process^11^,^12^, puts forward the claim that the deep knowledge about the localization of this actinide is mandatory, due to its environmental concern in the mining and metallurgical industry. However, the exact nature of this actinide element in the above natural (parent bauxite) and synthetic (bauxite metallurgical residue/BR) materials has been rather unknown, until the present paper. Hence, the scope of this study was to provide a thorough study on Greek BR with new insights, giving emphasis to the solid-state characterization of metals and metalloids, particularly to the nature of Th -related to radioactivity- and, in addition, to the mobility of these elements into the Mediterranean seawater. To the best of our knowledge, this is the first time in the literature that the leachability of BR in seawater is demonstrated. Leaching tests with acetic acid, instead of typical toxicity characteristic leaching procedure (TCLP) or by other conventional sequential extraction procedure^29^ modified after Tessier *et al.* (1979)^30^, as recently proposed in the case of potentially hazardous elements in BR^31^, were specifically applied for Th, REEs, and selected high field strength elements/HFSE (Nb, Ta), in long intervals (2 weeks to 1 year). Similar leaching experiments were additionally performed using Greek bauxites for comparative reasons. It should be mentioned that the thorough investigation of actinides, and namely of Th, aiming at the chemical behavior of the contaminant in the environment, has never been carried out with regard to BR originating from Greece; previous studies in Japan^32^ and recently in China^29^,^33^ have reported the problem. It is notable that although Gu & Wang (2013)^29^ have studied Th-rich (88–257 μg g^−1^) red mud by conventional transmission electron microscopy (TEM) the “accessory” and/or “neo-formational minerals” hosting the actinide element have never been determined. Of note, the structural environment of Th, particularly by extended X-ray absorption fine structure spectroscopy (EXAFS), has not been documented in the literature so far as in the case of other metals sorbed by Australian BR^34^. The final perspective of the present paper is to prospectively try to contribute to one of the world’s largest chronic problems, concerning the reuse of mining and mineral-processing residues and industrial wastes e.g.^35^ affecting the future of sustainable development^36^. The criticality of global aluminum demand^37^,^38^, resulting in the expansion of the Al industry and relevant solid wastes, enhances the necessity for detailed studies on the chemical, structural and environmental characteristics of BR. In conclusion, we consider that the study of actinides, and namely Th, in alumina refineries’ products can redound to a sustainable supply of strategic metals and oxides, and thus contribute to a more sustainable “modus operandi”.

## Results & Discussion

### Bulk and microscale characterization

**Figure 1:**
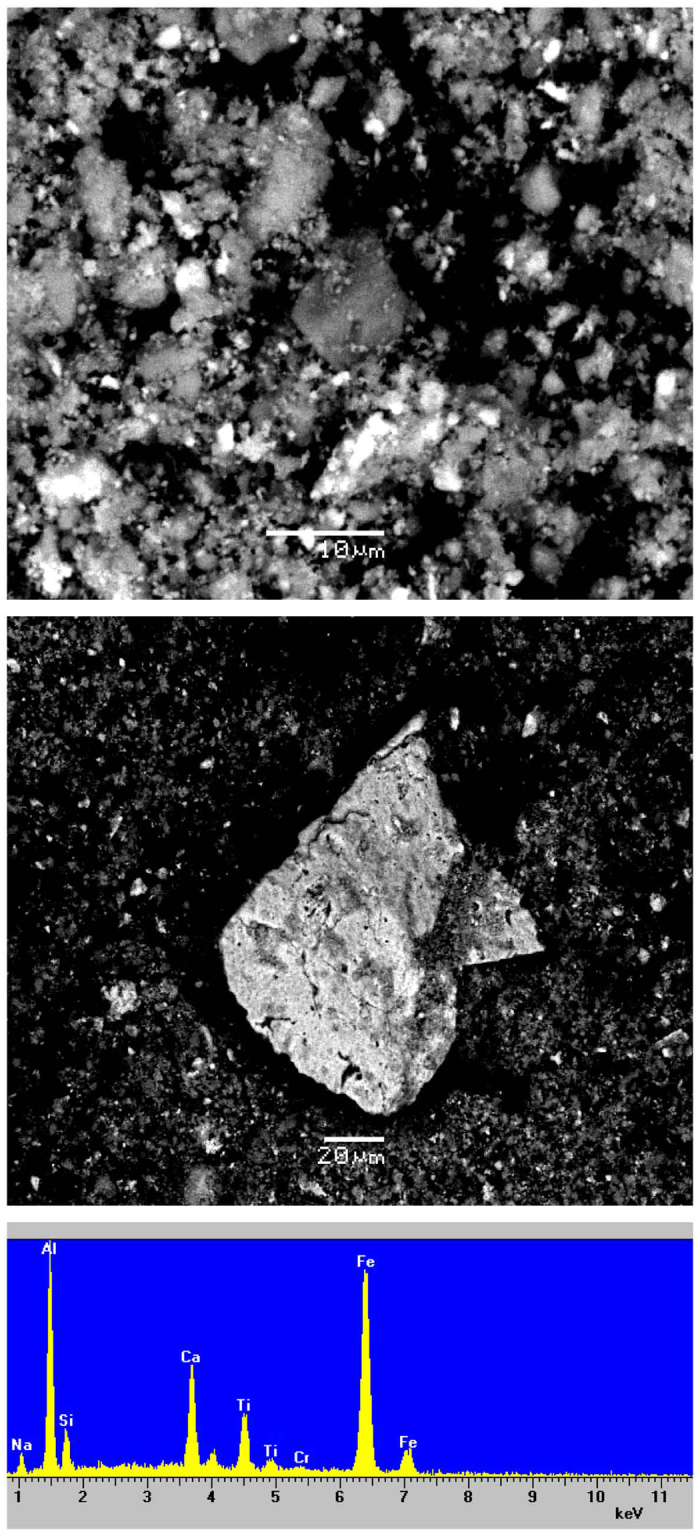
Morphology and chemical composition (major elements) of bauxite residue/BR (red mud) at the microscale, obtained by SEM-EDS, indicating the “Al-Fe-Ca-Ti-Si-Na-Cr matrix” which was further subjected to nanoscale study (see Figs 3 and 4).

Concerning the raw BR sample Fe, Al, Ca, Ti, Na, Si, O and H, are the elements corresponding to solids detected by powder X-ray diffraction/PXRD (see Supplementary Information and Supplementary Fig. S1), while phases related to other elements of interest, such as actinides (namely Th), Cr, V, As, Pb, as well as REEs, are not detectable. The same applies for the seawater- and the acid-treated BR samples. However, Cr, along with the above major elements, is additionally indicated by scanning electron microscopy (SEM) equipped with an energy dispersive spectrometry (EDS), into an “Al-Fe-Ca-Ti-Si-Na-Cr matrix” appearing in a few μm size (Fig. 1). Since all the micro areas of the raw sample, checked by SEM-EDS, follow the same spectral pattern and, therefore, it is argued that the raw BR is apparently rather homogeneous in terms of composition at the microscale. Taking into account the PXRD patterns, it could be assumed that this matrix is an admixture of nanocrystalline phases and potential amorphous and/or poorly crystalline phases.

The wavelength dispersive X-ray fluorescence (WDXRF) and, complementary, the inductively coupled plasma mass and optical emission spectroscopic measurements (ICP-MS/OES) of the studied BR proved that major Fe, Al, Ca, Si, Ti, Na and C, as well as significant volatiles (loss on ignition/LOI) comprise 86.5 wt.% and 13.6 wt.% of its chemical composition, respectively. In addition, when the trace elements’ concentrations of BR are normalized to the average values of Greek bauxites (*n *= 16)^28^ and of Upper Continental Crust (UCC) e.g.^28^ ^and references therein^, depletion in Ga (following the chemical behavior of Al) and enrichment -among others- in Cr (2403 μg g^−1^), V (1081 μg g^−1^), Ni (902 μg g^−1^), As (164 μg g^−1^), Pb (120 μg g^−1^), Sc (114 μg g^−1^) as well as in Cd, but also in Nb, Y, Ta, and REEs (Supplementary Table S3 and Supplementary Fig. S2) is pointed out. Moreover, high levels of Th (111 μg g^−1^) have been determined using high-resolution (HR) γ-ray spectroscopy; rather higher than those reported in the literature^28^,^39^ and references therein. The latter, and also minor U content (15 μg g^−1^), are responsible for the radioactivity of the studied BR (355 Bq kg^−1^ for ^232^Th and 133 Bq kg^−1^ for ^238^U) with a total dose rate of 295 nGy h^−1^ (see Supplementary Table S4). It should be mentioned that the radioactivity of Greek BR due to Th is in the same range from other BR reservoirs around the world, with the exception of the Australian BR, reaching up to 1900 Bq kg^−1^ (see Supplementary Table S4 and references therein). The studied BR has higher Th radioactivity compared to the average of the studied Greek bauxites from Parnassos-Ghiona mines (avg. 182 Bq kg^−1^; avg. total dose rate: 162 nGy h^−1^; *n* = 10). The same stands for radioactivity due to U, whereas both materials exhibit similar values due to ^40^K.

### Leaching experiments

**Figure 2:**
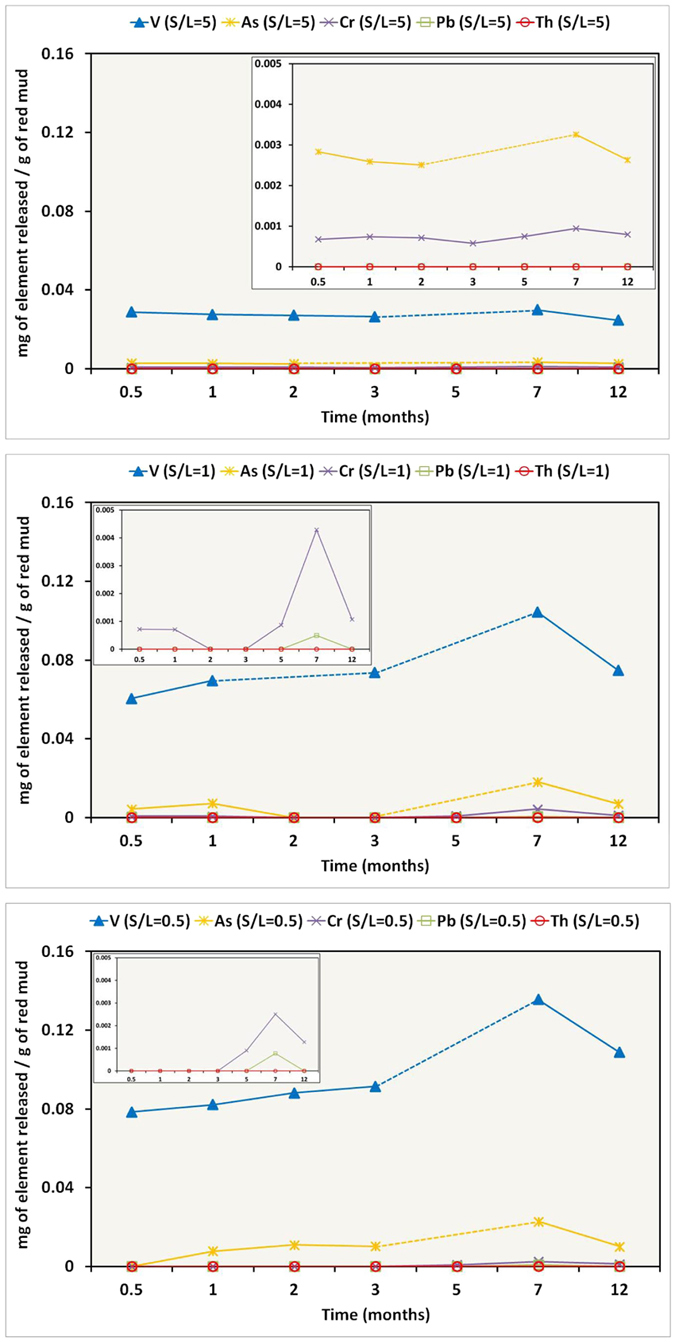
Results from leaching experiments of bauxite residue/BR (red mud) with Mediterranean seawater and variable S/L ratios, concerning V, As, Cr, Pb and Th.

Leaching experiments for the studied BR, in conjunction with sector field (SF) ICP-MS spectroscopy using Mediterranean seawater from Greece indicated significant release of V, relatively to seawater composition^40^, depending on S/L ratio (Fig. 2). Thus, V appears to be the most mobile element, perhaps due to its major association with rather soluble phases. This is in accordance with previous leaching NEN7341 tests concerning BR calcined in inert and reducing atmosphere^11^, as well as in good agreement with recent studies about V in Hungarian BR^1^,^7^. According to the latter studies, presenting X-ray absorption near-edge structure (XANES) spectroscopic data of vanadium (V *K*-edge), V in Hungarian BR is pentavalent associated with a Ca-Al-hydroxysilicate phase, corresponding to “hydrogarnet”-type phase. We can, therefore, assume that in Greek BR, having the same phase composition as the Hungarian BR, V is present most likely as VO_4_^3−^ anions, known to be mobile in seawater. On the other hand, V in Australian BR has been reported as tetravalent and/or trivalent related to ilmenite and/or goethite^41^. However, ilmenite has not been detected in Greek BR so far using PXRD, SEM or TEM (see text below), whereas the actual presence of goethite, either at the microscale or at the nanoscale, cannot be confirmed with certainty. Traces of As and Cr have also been detected, relatively to seawater composition^40^, after prolonged exposure in lower solid-to-liquid (S/L) ratio, while Pb is practically immobile. Based on previous Cr *K*-edge XANES and TEM studies^7^,^41^ Cr is associated with hematite showing less solubility. The most interesting point is that Th -related to radioactivity- seems to be immobile in seawater, at least after 12 months of leaching tests (Fig. 2). However, taking into account the PXRD study and the SEM-EDS investigation of the BR (see text above), it can be stated that there is no clear evidence of the Th-hosting phase(s) in the Greek BR at the microscale. That was finally realized combining the data yielded by both the scanning transmission electron microscopy-energy dispersive spectroscopy (STEM-EDS) and the electron energy-loss spectroscopy (EELS) together with the Th *L*_III_-edge EXAFS spectroscopic measurements on the studied BR (see text below). Analogous leaching experiments with the basic parent material (typical low-grade and high-grade Greek industrial bauxite) in Mediterranean seawater from Greece were carried out for comparative reasons; they showed negligible V, As, Cr, Pb, and Th release (Supplementary Fig. S3). The effect of S/L ratio is also depicted, indicating that lower S/L ratio causes a relative increase on the solubilization of heavy metals in the studied bauxites, during the experiments. Additional acetic acid-leaching experiments with the BR indicated significant release of V, as in the case of seawater, and much higher release of Cr (Supplementary Figs S4 and S5), a phenomenon also mentioned in the literature^31^. Nevertheless, it is worthy to note that the high release of REEs from BR (Supplementary Fig. S5), giving facts for potential recovery technologies inasmuch future availability of REEs is of great concern due to monopolistic supply conditions, environmentally unsustainable mining practices, and rapid demand growth^42^. This is in line with previous relevant works for Greek BR with regard to acid-recovery of lanthanides and Y^21^,^22^,^23^,^24^. In either case, the negligible effect of S/L ratio is also presented, indicating that the percentage of released elements -including the REEs- from leached BR is not severely affected by the S/L, during the experiment. Similar leaching experiments with typical low-grade (Fe-rich) and high-grade (Fe-depleted) Parnassos-Ghiona bauxites, presented for the first time in the literature, have also indicated significant recovery of REEs (Supplementary Figs S6 and S7). In the case of bauxites, the mobility of REE in acid is due to the presence of light REE (LREE) minerals (mostly bastnäsite/parisite-group) as reported previously^27^,^28^. Despite the fact that the mobility of Th in acid-treated bauxite residue is relatively enhanced, as compared to its negligible mobilization in seawater environment, considerable amount of this actinide element seems to remain in Greek BR. This robustly proves that the aforementioned acid-insoluble as well as seawater-insoluble solid nanophase (not apparent in the SEM-EDS observations) is hosting immobile Th into the “Al-Fe-Ca-Ti-Si-Na-Cr matrix”. The difference at the microscale between the initial (raw) and acid-treated “Al-Fe-Ca-Ti-Si-Na-Cr matrix” does affect mainly the lowering of Ca-*K*α X-ray emission peak in the EDS spectra (Fig. 1 and Supplementary Fig. S8) attributed to the loss of Ca-carbonate minerals and Ca-Al-hydroxysilicate phases.

### Nanoscale characterization

Except for the aforementioned observed solid phases at the microscale (see text above) calcium titanium oxide (CTO) phases are expected to be produced due to the addition of lime during the Bayer process^43^, under low-T and low-P conditions. Regarding CTO in Greek BR, a calcium titanium oxide phase previously identified by bulk PXRD^32^, was arbitrarily reported as “perovskite“^20^,^43^. However, perovskite was detected by PXRD in BR from Zhengzhou Changcheng alumina plant in China^43^,^44^, from the Aughinish alumina plant in Ireland^43^,^45^,^46^, and from India^43^,^47^. On the other hand, Santini (2015)^48^ in a similar study with regard to BR from USA, determined only sodalite- and cancrinite-phases but not any crystalline CTO and/or perovskite. Furthermore, conventional TEM “photographs” showed perovskite in BR from China^49^ without any structural proof.

**Figure 3:**
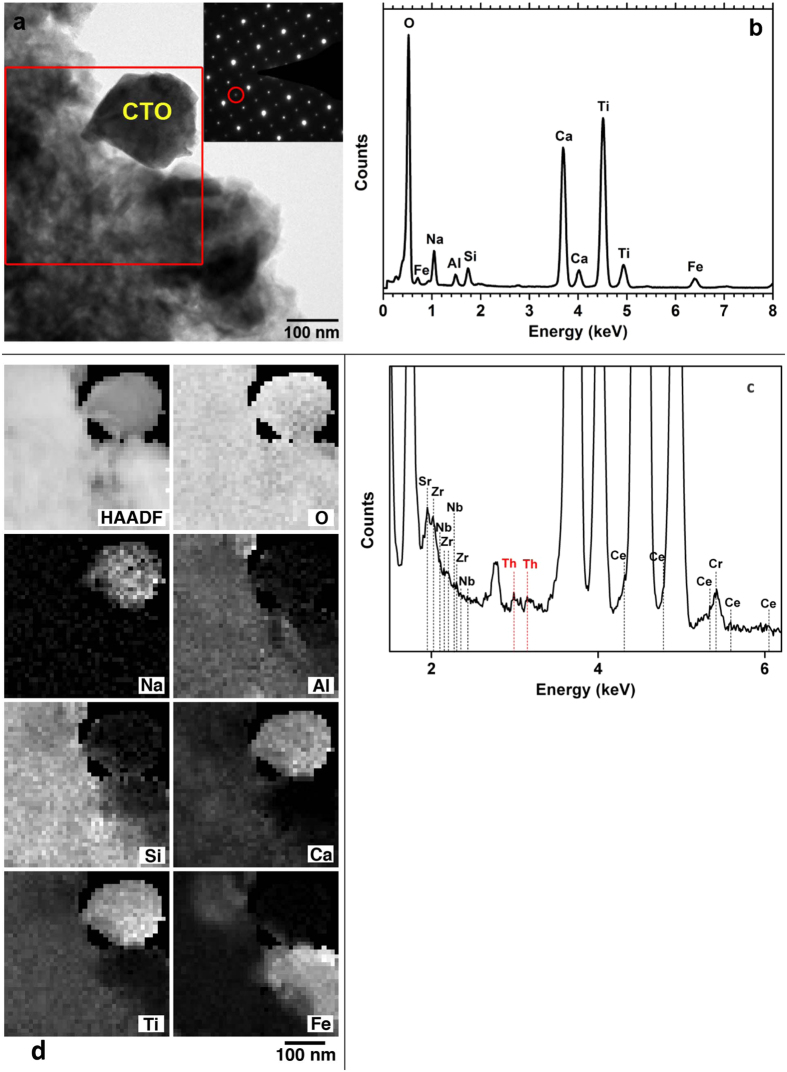
STEM-EDS data concerning a representative Th-hosting nano-perovskite (Ca_0.8_Na_0.2_TiO_3_/CTO) into bauxite residue (BR). Bright Field (BF) image and SAED pattern (**a**); STEM-EDS spectrum (**b**); enlarged energy area of the spectrum showing the presence of Th and minor elements (**c**). The viewing direction for the acquisition of SAED pattern is [101]. A peak at 452 eV is due to Ti Lα, while a second peak at 2.77 KeV and a third peak at 1.95 keV are attributed to the Si escape peak for Ti Kα and for Ca Kα, respectively; the latter one overlaps with the Sr, Nb and Zr peaks (**c**). The red-colored rectangle in the BF image indicates the area where the STEM-HAADF image and the EDS elemental maps were recorded. Superlattice reflections at the SAED pattern are marked with red-colored circle. STEM-HAADF image and EDS elemental maps (grayscale range of O: 0–62%; Na: 0–6%; Al: 0–17%; Si: 0–21%; Ca: 0–16%; Ti: 0–24%; Fe: 0–51%) for nano-perovskite crystallite (**d**). Fe is attributed to the neighboring Ti-containing hematite and the clay-like phases (see also Supplementary Fig. S11).

**Figure 4:**
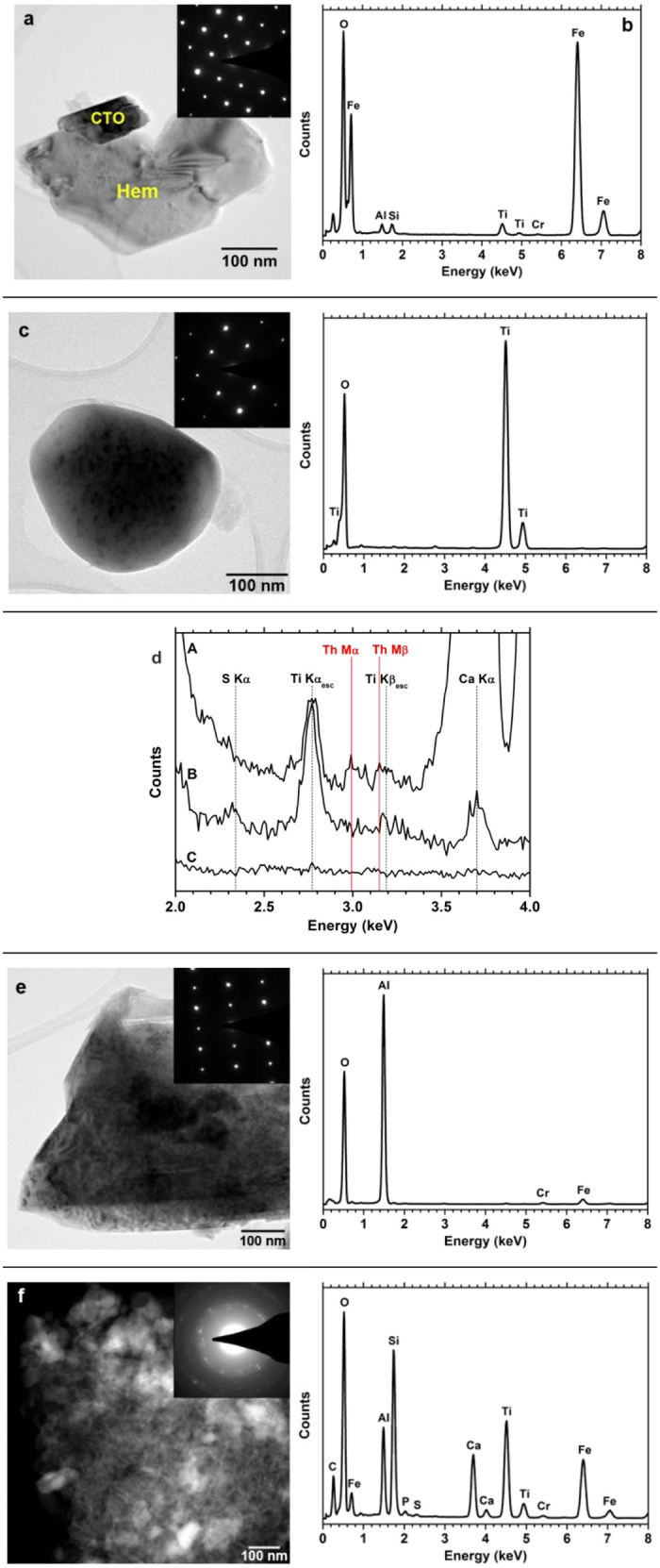
STEM-EDS data for Ti-containing hematite (Hem) particles intergrown with nano-perovskite (CTO). BF image and SAED pattern (acquired from [211] viewing direction; **a**) and the corresponding STEM-EDS spectrum (**b**, see also Supplementary Fig. S9). The almost visible narrow peak of Na is due to background noise. STEM-EDS data for anatase particle, including a BF image and SAED pattern as well as a STEM-EDS spectrum (**c**). The viewing direction for SAED pattern is [111]. A peak at 2.77 KeV is related to the Si escape peak for Ti Kα, while a peak at 3.19 KeV can be assigned as the Si escape peak for Ti Kβ. Comparison (**d**) between the STEM-EDS spectra of Th-hosting nano-perovskite (**a),** Th-free anatase (**b**) and Th-free Ti-containing hematite (**c**). The Th Mα and Th Mβ peaks are demonstrated (red lines) together with the STEM-EDS artefacts of the escape peaks for Ti Kα (2.77 KeV) and Ti Kβ (3.18 KeV). The theoretical intensity ratio of Th Mα to Th Mβ is 5:3. A peak at 2.34 KeV KeV may be associated with S (S Kα), while a peak at 3.7 KeV is assigned to Ca (Ca Kα). STEM-EDS data for AlOOH phase with a diaspore structure, including a BF image and a SAED pattern as well as a STEM-EDS spectrum (**e**). The viewing direction for its SAED pattern is [100]. STEM-EDS data for Th-free clay-like phases, including a BF image and its Debye-Scherrer ring patterns as well as a STEM-EDS spectrum (**f**). A peak at 2.77 KeV is related to the Si escape peak for Ti Kα. The almost visible narrow peak of Na is due to background noise.

According to the STEM-EDS/EELS study (Figs 3 and 4 and Supplementary Figs S9–S12), the most motivating of the Ti-phases constituting the microscale “Al-Fe-Ca-Ti-Si-Na-Cr matrix” of the Greek BR is an unusual low-T and low-P perovskite-type phase e.g.^50^ occurring in nanoscale (Fig. 3a,b). The major composition of this nano-perovskite has been determined by TEM-EDS to be Ca_0.8_Na_0.2_TiO_3_. Since several other minor elements (such as Ce, Nb, Zr, Cr and, possibly, Sr) might additionally be hosted as impurities (Fig. 3c) that might lead us to argue about a Ca-Na-(Ce-Nb-Zr-Cr)-nano-perovskite. This argument implies a minor contribution of Ce-loparite-type phase (Ce(Ti,Nb)O_3_). The most important finding is that this nano-perovskite contains Th (Fig. 3c), which is estimated to be 700 μg g^−1^. The STEM imaging, generated by the high angle annular dark field (HAADF) detector, together with the STEM-EDS mapping (Fig. 3d), clearly showed the distribution of key elements (such as Na, Ca, Ti, and O) for the Th-hosting nano-perovskite (CTO) that co-exists with other neighboring mineral phases (hematite and clay-like phases; see text below) into the “Al-Fe-Ca-Ti-Si-Na-Cr matrix”. In the depicted EDS maps Fe is exclusively attributed to the neighboring hematite, while Al is due to the clay-like phase. All the obtained selected area electron diffraction (SAED) patterns from nano-perovskite correspond to a perovskite structure [lattice parameters are a: 0.562 ( ± 0.02) nm, b: 0.752( ± 0.02) nm and c: 0.561 ( ± 0.02) nm, *Pnma*) with superlattice reflections (Fig. 3a)]. Since these reflections do not exist in the conventional perovskite we assume that they might be associated with the substitution of Na on A-site. It has recently reported that Na may incorporate into A-site of a ABO_3_ and BST perovskite, when synthesized at high-T or at high-P^51^,^52^,^53^, but that has never been observed at low-T and low-P perovskites. According to recent discussion by Seki *et al.* (2014)^53^, Na-ions could be linked with tilting of TiO_6_ octahedra, causing significantly stronger superlattice reflections in Ca-containing perovskites. Additional structural information on the novel Ca_0.8_Na_0.2_TiO_3_ is obtained by EELS measurements (see Supplementary Information and Supplementary Fig. S9). As it has already been mentioned, the BR nano-perovskite occurs together with Th-free Ti-containing hematite particles (Fig. 4). The concentration of Ti in this Ti-containing hematite has been measured up to 5 wt.%. Small amounts of Si, Al, Cr (Fig. 4b) and, probably, P (Supplementary Fig. S10) also exist in the Ti-containing hematite. Distinct Th-free Ti-oxides were characterized as anatase (Fig. 4c and Supplementary Fig. S11). The anatase crystallites exhibit rounded shape with a smooth surface. When the STEM-EDS spectra of Th-free Ti-containing hematite and anatase are compared with the EDS spectrum of the Th-containing nano-perovskite the total absence of Th is obvious (Fig. 4d). Concurrently, EDS artifacts like escape peaks for Ti Kα and Ti Kβ are also present. The peak ratio of Th Mα and Th Mβ was found to be about 5:3, which is in good agreement with the experimental profile, although the second broad peak (3.15 KeV) may be partially affected by the Ti Kβ escape peak at 3.18 KeV (Fig. 4d). Besides, several planar defects, common found in anatase, have been demonstrated by high-resolution TEM (HRTEM) imaging (**a**,**b** of Supplementary Fig. S11). Subsequently, the STEM-HAADF and HR-STEM-HAADF observations (see Supplementary Fig. S11c,d, respectively) have proved, along with the STEM-EDS spectra, the absence of Th in all studied anatase particles. The observed AlOOH polymorphs corresponding to diaspore (α-AlOOH) contain Fe and Cr impurities (Fig. 4e). Moreover, Th-free clay-like phases (with major d-spacings corresponding to 0.31 nm, 0.28 nm, 0.26 nm and 0.17 nm; Fig. 4f) have also been confirmed as components in the “Al-Fe-Ca-Ti-Si-Na-Cr matrix”. The obtained Debye-Scherrer ring patterns of clay-like particles suggest that this phase could be related to a zeolite-type material, which is rather doubtful due to its unusual chemical composition (Si: 19.87 wt.%; Al: 10.71 wt.%; Ca: 10.94 wt.%; Ti:20.63 wt.%; O: 37.81 wt.% as major elements and C, P and S as minor elements). We might also assume that this phase could be an admixture of several nano-phases. Iron and chromium peaks attributed to the presence of Ti-containing hematite crystallites, as these Fe-oxides, are often associated with clay-like phases and nano-perovskite into the “Al-Fe-Ca-Ti-Si-Na-Cr matrix” (Supplementary Fig. S12). Thus, the STEM-EDS/EELS study revealed that both the initial and acid-treated “Al-Fe-Ca-Ti-Si-Na-Cr matrix”, as appeared at the microscale, are actually an aggregate of several Al-, Fe-, Ca-, Ti-, Si-, Na-, Cr- (and also S-, P-, Ce-, Nb-, Zr-, and maybe, Sr-) particles, including the Th-hosting nano-perovskite. There is no evidence for other phases hosting Th, neither at the microscale (see text above) nor at the nanoscale, except for the nano-perovskite, but the minor presence of element traces, in isolated parts of various BR phases, might not be excluded.

### EXAFS spectroscopy and the local environment of Th

**Figure 5:**
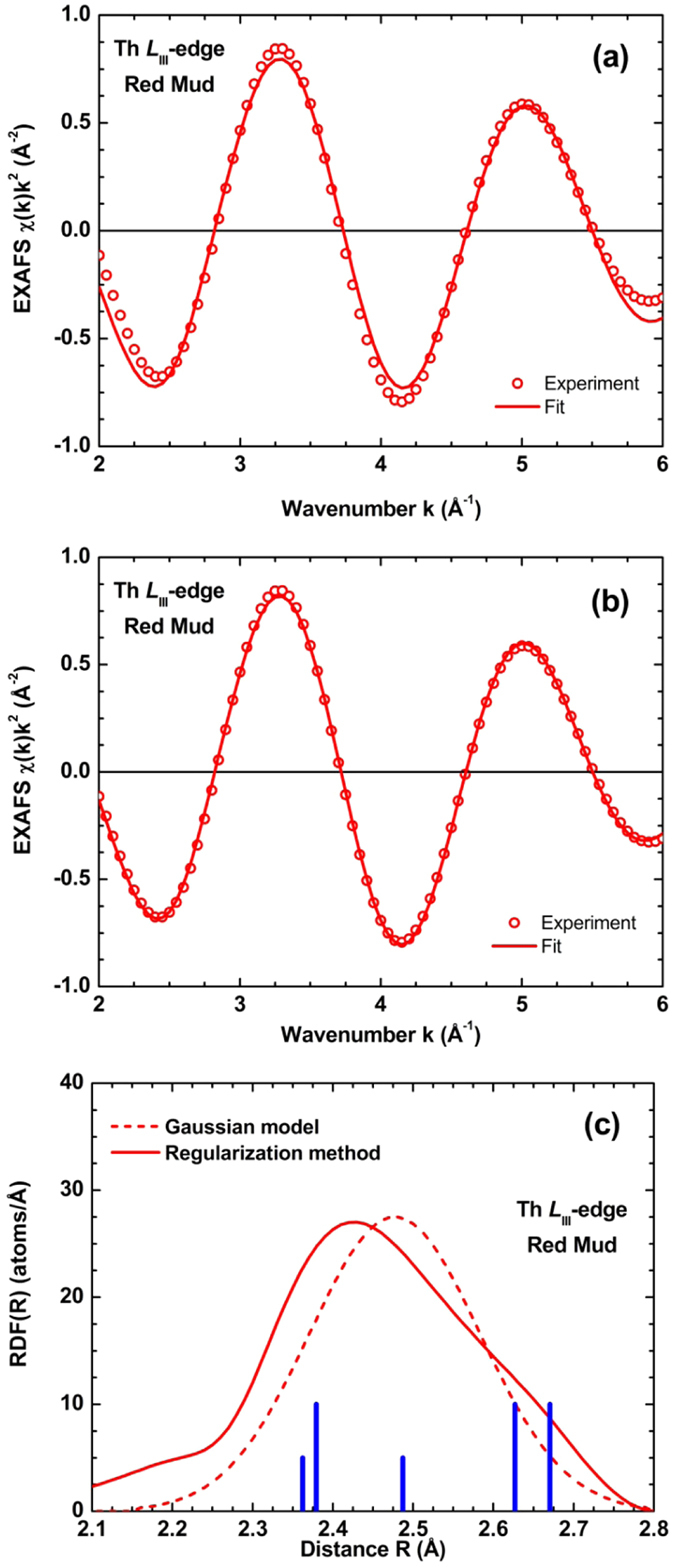
Best-fit results for the first shell Th *L*_III_-edge EXAFS in the Greek bauxite residue/BR (red mud) using the one-shell Gaussian model (**a**), and the regularization method (**b**). Comparison of the radial distribution functions (RDF’s), obtained from the first shell Th *L*_III_-edge EXAFS (**c**) using the one-shell Gaussian model (dashed line) and the regularization method (solid line). The blue bars indicate the position of the Ca-O distances in CaTiO_3_ perovskite^60^.

**Figure 6:**
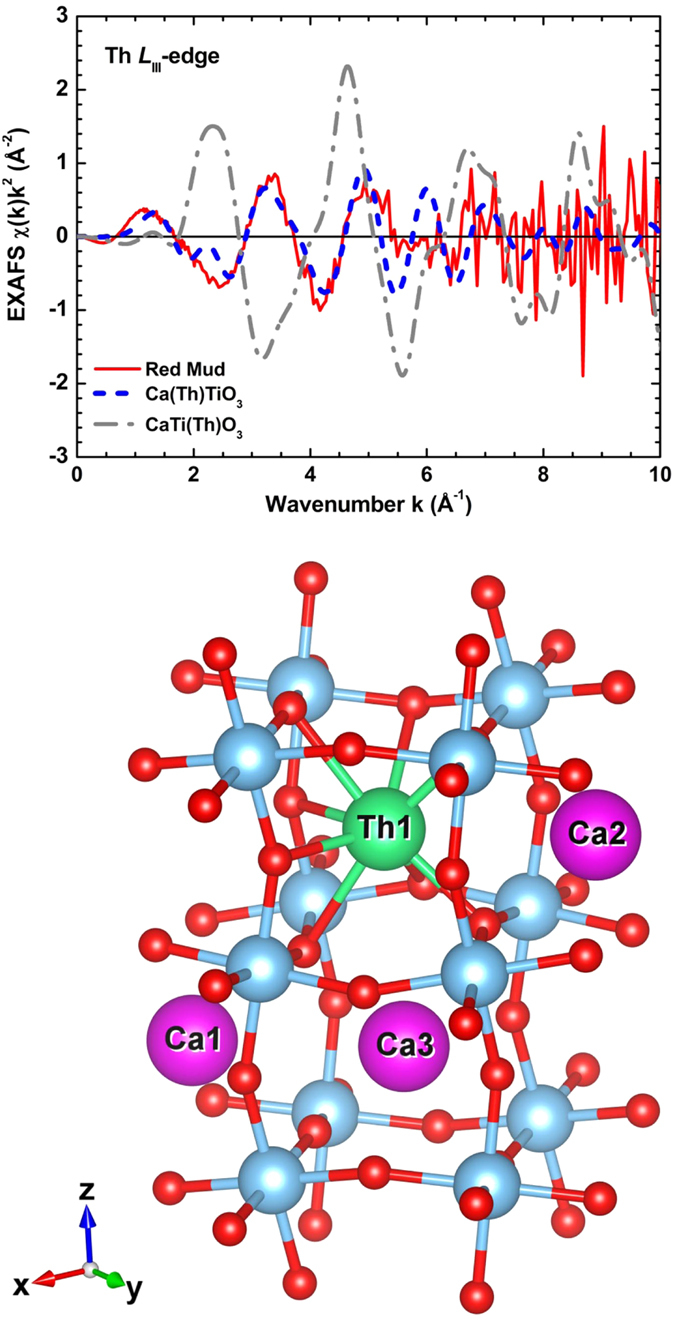
*Upper image*: The experimental (red solid line) and the calculated (blue dashed line: Th at the Ca site; grey dash-dotted line: Th at the Ti site) Th *L*_III_-edge EXAFS spectra of the studied Greek bauxite residue/BR (red mud) and Th-substituted CaTiO_3_ perovskite, respectively; *Lower image*: The structure of orthorhombic CaTiO_3_ perovskite^60^ with Th substituting Ca. Oxygen, titanium, and calcium atoms are illustrated by red-, blue-, and magenta-colored balls, respectively, whereas the thorium atom is indicated by green-colored ball.

Details on the structural characteristics of Th, assigned to the discovered low-T and low-P nano-perovskite (Ca_0.8_Na_0.2_TiO_3_), have been obtained by X-ray absorption fine structure spectroscopy/XAFS (Th *L*_III_-edge XANES and, especially, EXAFS) spectra and data processing using the ATHENA^54^ and EDA^55^ software packages. The Th *L*_III_-edge bulk XANES results of the studied BR, together with bulk & micro-XANES of Greek industrial bauxite^27^,^28^ in comparison with spectra of reference materials indicated that the valence of Th is typical 4+ (Supplementary Fig. S13). The experimental Th *L*_III_-edge EXAFS spectrum of the BR, which was measured in the fluorescence mode, is noisy above k = 6 Å^−1^ (Supplementary Fig. S14). This fact limits the accuracy of the analysis, as well as it reduces its resolution in the R-space. The EXAFS spectrum does not show any evidence for significant high frequency contributions either. This fact is compatible with the shape of the EXAFS spectrum Fourier transform (Supplementary Figure S14), which consists of a single broad peak located at 1.6 Å. Thus, the contribution of outer coordination shells around thorium is smeared out, and only analysis of the nearest environment can be performed. The first peak contribution into the total EXAFS spectrum was singled out by the Fourier filtering procedure and best fitted using two different approaches: the one-component Gaussian model^56^ and the regularization method^57^. It is considerable that in the latter method, the radial distribution function (RDF) could have had an arbitrary shape and, thus, may possibly account for anharmonicity and strong disorder effects. In both models, the theoretical backscattering amplitude and phases shift functions for the Th-O atom pair were calculated by the *ab initio* real-space FEFF8 code^58^, employing a complex Hedin-Lundqvist exchange-correlation potentially accounting for inelastic effects. The results of the best fits, obtained within the two models (i.e., Gaussian and regularization method) in the k-space range from 2 to 6 Å^−1^, are shown in the upper and middle images of Fig. 5, respectively, and the corresponding RDF’s of them are given in the lower image of Fig. 5. The regularization method results in better agreement, suggesting a deviation of the RDF shape from the Gaussian form. The numerical values of structural parameters for both models are given in Table 1. For the sake of discussion, it is noteworthy that, due to the short k-range interval of the EXAFS spectrum, there is a strong correlation between the values of the coordination number N and the Debye-Waller factor σ^2^. The local environment of Th ions in the studied BR is not very far from that in Fe-depleted bauxite^27^,^28^, but is significantly more disordered as is evidenced by the larger value of Debye-Waller factor in the Gaussian model and broad RDF’s in Fig. 5. As in the case of Fe-depleted bauxite^27^,^28^ the Th ions in BR are coordinated by about 7–8 oxygen atoms. This coordination number of Th is smaller than that in Th-nitrate (N = 12), being in agreement with the difference in the mean Th-O distances. However, it is notable that in the case of bauxite, being the basic parent material of BR, Th is hosted in microscale anatase (TiO_2_ polymorph) and there is no evidence for any perovskite phase. It is known that Th^4+^ in the structure of CaSiO_3_ perovskite, synthesized at high temperature (and occasionally at high pressure), may fundamentally occupy “large” Ca dodecahedral sites (^[12]^Ca^2+^) or “small” Si octahedral sites (^[6]^Si^4+^)^59^. On the other hand, in the structure of CaTiO_3_ perovskite^60^ Ti appears as ^[6]^Ti^4+^, whereas Ca may be ^[8]^Ca^2+^ or even ^[7]^Ca^2+^, compared to ^[12]^Ca^2+^ in the ideal perovskite structure. As one can see in the lower image of Fig. 5, the distribution of the Ca-O distances in CaTiO_3_ perovskite agrees well with the shape of the Th-O RDF in BR, obtained by the regularization method. Consequently, the Th *L*_III_-edge EXAFS signal was simulated by the FEFF8 code for the case of Th-absorbing atom substituting calcium or titanium in CaTiO_3_ perovskite. The calculated Th *L*_III_-edge EXAFS signals are compared with the experimental one for BR; see upper image of Fig. 6). In the frame of this simulation, the atom positions were fixed as in orthorhombic CaTiO_3_ perovskite-type structure^60^, and all Debye-Waller factors were set to zero that explains smaller damping of the calculated EXAFS amplitude at larger k-values. As one can see (upper image of Fig. 6), the model of Th at the Ca site (Ca(Th)TiO_3_) results in the overall amplitude and main frequency of the EXAFS signal close to the experimental one, whereas the model of Th at the Ti site (CaTi(Th)O_3_) differs from the experiment significantly, in both amplitude and frequency. Unfortunately, the weak contribution of the outer shells (Supplementary Fig. S14) in the experimental Th *L*_III_-edge EXAFS spectrum in BR does not allow us to make unambiguous conclusion on the Th location. However, taking into account the above argument about Th in Ca site of the nano-perovskite, in conjunction with the best-fit EXAFS results (R = 2.42–2.48 Å and CN = 7–8), we could presume that Th, hosted in the low-T and low-P novel Ca-Na-(Ce-Nb-Zr-Cr)-nano-perovskite of the studied BR, occupies Ca^2+^ sites rather than Ti^4+^ sites (lower image of Fig. 6).

### Environmental and technological implications about the Greek bauxite residue (red mud)

It is herein stated that the novel low-T and low-P Th-hosting Ca-Na-(Ce-Nb-Zr-Cr)-nano-perovskite (Ca_0.8_Na_0.2_TiO_3_) is the reason of the low Th release in acid medium, and subsequently of the Th immobility if the Greek bauxite residue/BR (i.e., red mud) is exposed to Mediterranean seawater. In general, it is demonstrated that Greek BR, and perhaps huge quantities of BR around the world, do not consitute, under certain circumstances an environmental hazard due to actinide content and radioactivity. In turn, BR is a valuable alumina refineries’ by-product to be used as secondary resource for a sustainable supply of critical & strategic elements (such as REEs) and, thus contribute to a more sustainable “modus operandi”.

## Conclusions

The nature of thorium in the bauxite residue/BR (the so-called “red mud”) from Greek Al industry has been investigated in detail for the first time in the literature. The interest arises from the fact that huge quantities of that BR, showing relative radioactivity, had been deposited into the Mediterranean Sea in Greece. The chemical analysis and the HR γ-ray measurements proved that the observed higher radioactivity is higher compared to the parent Greek bauxite, mainly attributed to the presence of Th (111 μg g^−1^; 355 Bq kg^−1^ for ^232^Th). The marine environment might not be affected by this actinide element; leaching experiments have confirmed negligible Th release in Mediterranean seawater, at least after 12 months of interaction. In contrast, the experiments of the present study indicated that the mobility of V might be of potential risk. Characterizing the studied BR in microscale yielded no evidence of Th hosting phase into a homogeneous “Al-Fe-Ca-Ti-Si-Na-Cr matrix. However, the STEM study of the leached BR sample at the nanoscale showed that the immobility of Th can be attributed to the existence of an insoluble nano-perovskite with major composition of Ca_0.8_Na_0.2_TiO_3_. Subsequent study of Th *L*_III_-edge EXAFS spectroscopy revealed that the local environment of Th^4+^ in the structure of nano-perovskite is occupying Ca^2+^ sites, rather than Ti^4+^ sites. We do consider that this structural peculiarity is related to the negligible Th release in the Mediterranean seawater.

## Materials and Methods

### Samples and Initial Characterization (PXRD, WDXRF, ICP-OES/MS, HR γ-ray spectroscopy)

The BR samples were supplied by the “Aluminium of Greece S.A.” alumina plant at Agios Nikolaos (Antikyra, Gulf of Corinth, central Greece). The WDXRF measurements for the major and trace elements of BR were performed on a PANalytical AxiosmAX WDXRF spectrometer using the Pro - Trace measurement and analysis application package at the PANalytical B.V. laboratories. For more details about the samples and about the characterization techniques, please consult the Supplementary Information.

### Seawater and Acid-Leaching Experiments (SF-ICP-MS)

Leaching experiments on the investigated BR and bauxite samples were carried out using (a) Mediterranean seawater from the Gulf of Corinth (Greece), and (b) concentrated acetic acid (Merck), over a period ranged from 2 weeks to 1 year. Acetic acid was used, instead of typical TCLP, according to a recent relevant work on red mud^31^. The analyses of leachates for potentially hazardous metals and metalloids (Cr, V, Ni, As, Pb), Th, Ta, Nb, and REE, were performed using a SF-ICP-MS (Thermo Scientific Element 2/XR). For more details, please consult the Supplementary Information and the Supplementary Tables S1 and S2.

### Electron Microscopy (SEM-EDS, STEM-EDS/EELS and STEM-HAADF)

Details on the elemental composition of BR were obtained on carbon-coated free surfaces and polished (in epoxy resin) solid samples using a Jeol JSM-5600 SEM equipped with an Oxford EDS. The observations were made with an accelerating voltage of 20 kV, a working distance of 20mm, a current beam of 1.5 nA, an active time of 20–100 s and a magnification from ×35 to ×3500. Pure metallic materials and minerals were used as standards. STEM-EDS/EELS and STEM-HAADF were carried out using an FEI probe-corrected Titan 80-300ST FEG TEM, equipped with an Oxford Instrument X-Max 80 mm^2^ silicon drift detector and a Gatan Tridium imaging filter. SAED was used to identify mineral phases. For the detection of Th, we performed TEM-EDS with count rates of 5,000–8,000 counts sec^−1^ and dwell times of 60–180 sec (i.e., at total counts of 500,000–700,000), using a Gatan low-background Be specimen holder. The EELS measurements were made in TEM diffraction mode with an energy resolution of 0.9 eV.

### X-ray Absorption Fine Structure Spectroscopy (XAFS)

Bulk XAFS (EXAFS/XANES) spectroscopic study of BR was performed at the Th *L*_III_-edge (16300 eV; energy was calibrated using a Y metal foil) in the fluorescence mode on powdered samples pressed with cellulose into pellets. Spectra were obtained at the SUL-X beamline of the ANKA Synchrotron Radiation Facility (KIT, Germany). Thorium compounds (Th(NO_3_)_4_ • 4H_2_O and ThO_2_) and minerals containing Th impurities, such as zircon (ZrSiO_4_), were used as reference materials. The XAFS spectra were analyzed using the ATHENA^54^ and the EDA^55^ software packages. The EXAFS signal corresponding to the first main peak in Fourier transforms (FTs) was isolated using the Fourier filtering procedure. The range of the back-FT was 1–3 Å for Th(NO_3_)_4_ • 4H_2_O and 0.8–2.5 Å for the samples. Thus, obtained EXAFS signals were simulated using two different approaches: the conventional Gaussian model^56^ and the regularization method^57^. The theoretical backscattering amplitude and phase shift functions for Th-O atom pair were calculated by the *ab initio* FEFF8 code^58^ using a complex Hedin-Lundqvist exchange-correlation potential accounting for inelastic effects.

## Additional Information

**How to cite this article**: Gamaletsos, P. N. *et al.* The role of nano-perovskite in the negligible thorium release in seawater from Greek bauxite residue (red mud). *Sci. Rep.* **6**, 21737; doi: 10.1038/srep21737 (2016).

## Supplementary Material

Supplementary Information

## Figures and Tables

**Table 1:** Th structural parameters for the studied Greek bauxite residue/BR (red mud), in comparison with the Greek bauxite values^27^,^28^, obtained from the processing of the EXAFS signals using the EDA software package^55^.

	Greek Red Mud (present study) Th *L*_III_-edge bulk-EXAFS	Greek Bauxite^27^,^28^	
		Th *L*_III_-edge bulk-EXAFS	Th *L*_III_-edge Micro-EXAFS
***Gaussian model***			
**CN** ± 0.7	7.3	6.9	6.9
**R (Å)** ± 0.04	2.48	2.46	2.45
**σ**^**2**^ **(Å**^**2**^) ± 0.002	0.011	0.007	0.006
***Regularization method***			
**CN**	8.0	7.4	7.4
R (Å)*	2.42	2.40	2.38

^*^This distance is the position of the RDF maximum.
