# Supplementary material for: The role of nano-perovskite in the negligible thorium release in seawater from Greek bauxite residue (red mud)
Source: Sci Rep. 2016 Feb 22;6:21737. doi: 10.1038/srep21737 (PMC4761986; doi:10.1038/srep21737)
Supplement: Supplementary Information [file srep21737-s1.pdf]

Supplementary Information

The role of nano-perovskite in the negligible thorium release in seawater from Greek bauxite residue (red mud)

Platon N. Gamaletsos[1,2,6,*], Athanasios Godelitsas[2], Takeshi Kasama[1], Alexei Kuzmin[3], Markus Lagos[4], Theo J. Mertzimekis[5], Jörg Göttlicher[6], Ralph Steininger[6], Stelios Xanthos[7,8], Yiannis Pontikes[9], George N. Angelopoulos[10], Charalampos Zarkadas[11], Aleksandr Komelkov[11], Evangelos Tzamos[12] and Anestis Filippidis[12]

[1] Center for Electron Nanoscopy, Technical University of Denmark, 2800 Kongens Lyngby, Denmark

[2] Faculty of Geology & Geoenvironment, National and Kapodistrian University of Athens, Zografou Campus, 15784 Athens, Greece

[3] Institute of Solid State Physics, University of Latvia, Kengaraga str. 8, 1063 Riga, Latvia

[4] Karlsruhe Institute of Technology, Institute for Nuclear Waste Disposal, Hermann-von-Helmholtz-Platz 1, 76344 Eggenstein-Leopoldshafen, Germany

[5] Faculty of Physics, National and Kapodistrian University of Athens, Zografou Campus, 15784 Athens, Greece

[6] Karlsruhe Institute of Technology, ANKA Synchrotron Radiation Facility, Hermann-von-Helmholtz-Platz 1, 76344 Eggenstein-Leopoldshafen, Germany

[7] Department of Electrical and Computer Engineering, Nuclear Technology Laboratory, Aristotle University of Thessaloniki, 54124 Thessaloniki, Greece

[8] Department of Automation Engineering, Alexander Technological Educational Institute of Thessaloniki, 57400 Thessaloniki, Greece

[9] KU Leuven, Department of Materials Engineering, Kasteelpark Arenberg 44, 3001 Leuven, Belgium

[10] University of Patras, Department of Chemical Engineering, 26500 Rio, Greece

[11] PANalytical B.V., 7600 AA Almelo, The Netherlands

[12] School of Geology, Aristotle University of Thessaloniki, 54124, Thessaloniki, Greece

[*] Corresponding author: Platon Gamaletsos (plagka@dtu.dk)

This supplement contains:

1. Supplementary Figures (14)

2. Supplementary Tables (4)

3. Supplementary References (27)

Materials and Methods

Samples. The bauxite residue / BR (red mud) samples were supplied by the "Aluminium of Greece S.A." alumina plant at Agios Nikolaos (Antikyra, Gulf of Corinth, central Greece). The Company manages BR through the installation of four filter presses, disposing its final dewatered metallurgical residue (so-called "red mud") onto a special configured area inside its plant. The bauxite residue, used in this study, consisted of composite sample, which was created by taking the appropriate number of specimens (*n*=10) and using standard mixing procedures and sample splitters. Moreover, basic parent material (bauxite) samples (*n*=16), used in the present study for reasons of comparison, were collected from underground mining sites at the Parnassos-Ghiona, which is an active mining area of the three Greek mining companies ("Aluminium of Greece S.A." and its subsidiary "Delphi-Distomon S.A.", "S&B Industrial Minerals S.A." that has been recently consolidated by "Imerys S.A.", and "ELMIN Hellenic Mining Enterprises S.A."), exploiting bauxite from the Parnassos-Ghiona area (Central Greece). Greek industrial bauxites actually represent the typical low-grade (i.e., Fe-rich; diasporic & boehmitic; red-brown in color) and the high-grade (i.e., Fe-depleted; diasporic; white-grey in color) exploitable samples. It is worth noting that, except for Greek karst-type bauxite, which is the major part of the parent material that "Aluminium of Greece S.A." uses at its industrial plant, the company also imports a small proportion of tropic bauxite for its needs, in order to be used as a mixture of Al-ores for its alumina production. On this basis, since the major part of parent material does consist of Greek bauxites, we strongly claim that karst-type bauxites from Parnassos-Ghiona active mining area represent the basic parent material of the present work.

Powder X-ray Diffraction (PXRD) and Bulk Chemical Analyses (WDXRF and ICP-OES/MS). The PXRD characterization of the raw, seawater-leached and acetic acid-leached BR sample was carried out using a Siemens D5005 (currently Bruker AXS) diffractometer with CuK_{α} radiation (λ = 1.54 Å) at an accelerating voltage of 40 kV. Along with the major and trace element chemical analyses that were performed at the PANalytical B.V. laboratories (using a PANalytical AxiosmAX WDXRF spectrometer), complementary analyses were also performed using a Perkin Elmer ICP-OES and a Perkin Elmer Sciex Elan 9000 ICP-MS spectrometer following a $LiBO_2/LiB_4O_7$ fusion and HNO_3 digestion of a 0.2 g sample. In addition, a separate 0.5 g split was digested in a HNO_3:HCl mixture (1:3) and analyzed by ICP-MS for precious and base metals.

High-Resolution (HR) Gamma-Ray Spectroscopy. HR γ-ray spectra of BR and the bauxite samples were collected using a Canberra high-purity germanium (HPGe) detector. Spectra analysis resulted in the natural radioactivity levels and total dose rate.

Seawater and Acid-Leaching Experiments (SF-ICP-MS). The bauxite residue / BR (red mud) used in the leaching experiments is a composite sample which was created by taking the appropriate number of specimens (*n*=10), using standard mixing procedures and sample splitters. Simultaneously, similar leaching tests on Greek industrial bauxites were also carried out for comparison. For this purpose, two composite samples were also created to be used at leaching experiments, too. The bauxite composited samples, which are obtained from bauxite mines of "Aluminium of Greece S.A." and its subsidiary "Delphi-Distomon S.A.", represent both the typical low-grade (i.e., Fe-rich; diasporic & boehmitic; red-brown in color) and high-grade (i.e., Fe-depleted; diasporic; white-grey in color) karst-type bauxites from the Parnassos-Ghiona active mining area (central Greece). On a first step, powder from the BR, as well as from both the karst-type of bauxites (typically between ~ 10 and 100 mg; details are given in **Supplementary Table S1**) were weighted in zinser vials, which were then filled with 20 mL of the leaching solution (i.e., seawater or concentrated acetic acid). In addition, two experimental blank runs (only seawater and concentrated acetic acid) were also prepared in the same way using zinser vials filled with 20 mL of the particular solution. Sampling of the experimental, as well as the blank solutions, was performed at certain periods of time after the start of the leaching procedure (after 2 weeks, 1 month, 2 months, 3 months, 5 months, 7 months, 10 months, 12 months). 1 mL was taken from each vial and filtered with a syringe filter to remove particles. 500 µL of the filtered leachate was then further diluted using 2 vol% HNO_3 (prepared from concentrated Merck ultrapure HNO_3 and deionized water with a resistance of 18 mΩ) to a total of 5 mL. The concentrations of trace elements, including actinides, HFSE and REEs, in the leachates were determined using a SF-ICP-MS spectrometer (Thermo Scientific Element 2/XR). Lanthanides and As were measured in high resolution mode (m/Δm = 10,000), whereas other elements such as Cr, and V were measured in medium resolution (m/Δm = 4,000) or in case of Ta, Y, Th, and Pb in low-resolution mode (m/Δm = 300). Rhodium was added to the solutions and used as an internal standard (final Rh concentration was 1 ng g^{-1}). Plasma parameters and sample uptake conditions were optimized at the beginning of each instrumental session to achieve optimal signal stability and maximum sensitivity at low oxide ratios. Typical instrumental parameters and measured isotopes are listed in **Supplementary Table S2**. Calibrations were generally performed with adjusted solutions using acetic acid and Mediterranean seawater for matrix matching. The accuracy of the measurements was verified with various water reference materials (TM26.3 and SPS-SW1), which were also adjusted with Mediterranean seawater and acetic acid for matrix matching. Since the water reference materials are not certified for Ta a diluted matrix matched reference solution of BHVO-1 (rock reference standard material) was used. All certified values could be reproduced throughout the measurements except for Dy, which is therefore not listed in **Supplementary Table S2**. The analyzed

reference material solutions yielded consistently higher Dy concentrations due to unidentified spectral interferences.

Results and Discussion

PXRD study. According to the PXRD patterns (**Supplementary Fig. S1**) the investigated Greek BR contains hematite (α-Fe_2O_3), calcite ($CaCO_3$), AlOOH polymorphs (gibbsite: $Al(OH)_3$; diaspore; α-AlOOH) as well as Na-Ca-Al-silicate-carbonate and Ca-Al-hydroxysilicate phases (cancrinite- and “hydrogarnet”-type phases). “Hydrogarnet” may correspond to katoite[1] ($Ca_3Al_2(SiO_4)_{3-x}(OH)_{4x}$, x=1.5-3.0 or $\{Ca_3\}[Al_2](\square_3)(OH)_{12}$), on the basis of previous measurements[2]. It should be noted that the same crystalline phases have also been detected in BR from Ajka alumina plant accident[3]. The same phases have also been detected in the case of seawater-treated samples. On the other hand, carbonate minerals (at least calcite), and also Ca-Al-hydroxysilicate phases, are practically absent in BR samples leached with acetic acid solutions. There is also a severe decrease in the intensity of cancrinite ($Na_6Ca_2Al_6Si_6O_{24}(CO_3)_2$) peaks, in accordance with previous observations for acid-treated BR, by Liang et al. (2014)[4]. Moreover, distinct peaks of anatase, quartz and phyllosilicates/clay-like phases are revealed in the PXRD patterns of the acetic-acid leached BR. Therefore, Fe-oxides, Ti-oxides, AlOOH-polymorphs and clay-like phases are resistant in seawater and acetic acid solutions[4,5].

EELS Measurements. The Ti $L_{2,3}$-, Ca $L_{2,3}$- and O K-edge EEL spectral data (**Supplementary Fig. S9**) indicate that the phase found in Greek BR has rather similar spectral characteristics with CTO studied by Calvert et al. (2006)[6]. The comparison of the nano-perovskite Ti $L_{2,3}$- and O K-edges with CTO phases from literature has revealed that the Ti $L_{2,3}$-edges show structural similarities in terms of the observed crystal field effects[6]. In particular, the Ti $L_{2,3}$-edges show a well defined crystal field splitting of both the L_3 (A1 and A2 peaks) and L_2 edges (B1 and B2 peaks), very similar to the splitting phenomenon for reference $CaTiO_3$ perovskite phase[6]. On the other hand, the splitting of L_2 edge, into the B1 and B2 peaks seems to be less well-defined than that of L_3. The splitting can be assigned to a slight distortion of the TiO_6 octahedra from tilting, due to the presence of Ca and Na, and/or changes in the degree of covalence in Ti-O bonding in the studied phase. It can also be assumed that BR nano-perovskite contains only Ti^{4+}, having almost the same EEL spectrum to that of CTO from literature[6]. Furthermore, the O K-edges can be separated into the A_I and A_{II} components, corresponding to the A peaks that commonly appear within the 5-6 eV above the edge threshold; this is followed by another B peak, corresponding thus to B peaks that usually appear 10 eV above the threshold. The difference

is the small and narrow A2 peak, between the A1 and A* peaks of Calvert's reference CTOs[6], which is not obvious in our O *K*-edge spectrum, maybe due to the presence of the stronger A_{II} component within a few electron volt or due to poor signal-to-noise ratio in the spectrum. The broader A_{II} component of our novel CTO is close to the intensity of sharper A_{I} component, giving assumption of a higher energy-loss shoulder. The broad B peak resembles to that of A_{II} peak and does not show any splitting phenomenon, which agrees to that of Calvert's reference CTOs[6]. In general, the shape of the O *K*-edge spectrum is in agreement. Taking into account the above, one may say that A components of the O *K*-edge EEL spectrum are attributed to the hybridization of the O 2p with Ti and Ca 3d-like states, while the B peak is due to hybridization of O 2p with TM 4s- and continuum-like states, as mentioned for CTO phases[6]. Moreover, according to the Na *K*-edge EEL data, there is no doubt that our nano-perovskite also contains Na, which has been previously confirmed by STEM-EDS spectra (**Fig. 3**) and, additionally, by the quantitative EDS elemental maps (see image **d** of **Fig. 3**). Furthermore, the absence of N (401 eV) was confirmed as well, which "artificially" has been detected at 452 eV by STEM-EDS (see image **b** of **Fig. 3**). Actually, this "artificial" EDS N peak is attributed to Ti Lα.

Supplementary Table S1: Details of leaching experiments with regard to the studied bauxite residue / BR (red mud) and Greek typical low grade (i.e., Fe-rich; diasporic and boehmitic; red-brown in color) and high grade (i.e., Fe-depleted; diasporic; white-grey in color) bauxite samples.

Starting Date	Treatment Solution	Bauxite residue (red mud) & bauxite samples			Sample Weight (mg)	Amount of Solution (mL)
		Type		Code		
6.11.2012	Concentrated Acetic Acid	Red Mud		RM-AC-1	102.4	20
6.11.2012				RM-AC-2	12.5	20
6.11.2012		Greek Bauxite	High Grade	HGB-AC-1	99.1	20
6.11.2012				HGB-AC-2	11.8	20
6.11.2012			Low Grade	LGB-AC-1	104.2	20
6.11.2012				LGB-AC-2	9.2	20
6.11.2012	Mediterranean seawater (Gulf of Corinth – Greece)	Red Mud		RM-SW-1	98.9	20
6.11.2012				RM-SW-2	18.7	20
6.11.2012				RM-SW-3	9.1	20
6.11.2012		Greek Bauxite	High Grade	HGB-SW-1	102.7	20
6.11.2012				HGB-SW-2	20.9	20
6.11.2012				HGB-SW-3	10.8	20
6.11.2012			Low Grade	LGB-SW-1	99.2	20
6.11.2012				LGB-SW-2	19.9	20
6.11.2012				LGB-SW-3	9.9	20

Supplementary Table S2: Instrumental parameters for SF-ICP-MS measurements.

Instrumental settings	
Forward Power (W)	1250
Cooling gas flow rate (L min^{-1})	16
Auxiliary gas flow rate (L min^{-1})	0.9
Sample gas flow rate (L min^{-1})	1.195
Cones	Nickel
Sample introduction	
Nebulizer	PFA micro-concentric
Spray chamber	PC^3-System
Data acquisition	
Monitored masses	^{232}Th, ^{207}Pb, ^{208}Pb, ^{89}Y, ^{181}Ta (low resolution mode)<br>^{53}Cr, ^{51}V, ^{113}Cd (medium resolution mode)<br>^{139}La, ^{140}Ce, ^{141}Pr, ^{146}Nd, ^{149}Sm, ^{153}Eu, ^{158}Gd, Dy, ^{159}Tb, ^{165}Ho, ^{167}Er, ^{169}Tm, ^{173}Yb, ^{175}Lu, ^{75}As (high resolution mode)

Supplementary Table S3: Major and trace element concentrations in the studied bauxite residue (BR) mainly according to the WDXRF and, complementary to the ICP-OES/MS measurements. Commonly, the elements in the light REE (LREE) and heavy REE (HREE) groups are arbitrary defined[7-9].

**Fe_2O_3**	(wt.%)	41.27	**Cr**	(μg g^{-1})	2403	**Y**	(μg g^{-1})	97	**Gd**	(μg g^{-1})	16	**Br**	(μg g^{-1})	4
**Al_2O_3**	(wt.%)	15.90	**Zr**	(μg g^{-1})	1188	**Nd**	(μg g^{-1})	92	**Yb**	(μg g^{-1})	15	**Tb**	(μg g^{-1})	3
**CaO**	(wt.%)	12.99	**V**	(μg g^{-1})	1081	**Zn**	(μg g^{-1})	58	**U**	(μg g^{-1})	15	**Rb**	(μg g^{-1})	3
**SiO_2**	(wt.%)	6.12	**Ni**	(μg g^{-1})	902	**Cu**	(μg g^{-1})	54	**Sm**	(μg g^{-1})	15	**Lu**	(μg g^{-1})	2
**TiO_2**	(wt.%)	5.78	**Ce**	(μg g^{-1})	439	**Co**	(μg g^{-1})	40	**Te**	(μg g^{-1})	13	**Tm**	(μg g^{-1})	2
**Na_2O**	(wt.%)	2.86	**Ba**	(μg g^{-1})	234	**Ga**	(μg g^{-1})	39	**Er**	(μg g^{-1})	13	**Se**	(μg g^{-1})	2
**Total C**	(wt.%)	1.53	**As**	(μg g^{-1})	164	**Hf**	(μg g^{-1})	30	**Cs**	(μg g^{-1})	13	**Cd**	(μg g^{-1})	0.5
**LOI**	(wt.%)	13.6	**Sr**	(μg g^{-1})	131	**W**	(μg g^{-1})	30	**Ta**	(μg g^{-1})	8	**Ag**	(μg g^{-1})	0.3
**MgO**	(wt.%)	0.21	**Pb**	(μg g^{-1})	120	**Sb**	(μg g^{-1})	26	**Ge**	(μg g^{-1})	8	**Hg**	(ng g^{-1})	40
**P_2O_5**	(wt.%)	0.12	**La**	(μg g^{-1})	115	**Pr**	(μg g^{-1})	24	**Bi**	(μg g^{-1})	7	**Au**	(ng g^{-1})	bdl
**K_2O**	(wt.%)	0.08	**Sc**	(μg g^{-1})	114	**Dy**	(μg g^{-1})	20	**Be**	(μg g^{-1})	5	**ΣLREE*[1]**	(μg g^{-1})	**704**
**Total S**	(wt.%)	0.07	**Th**	(μg g^{-1})	111	**Sn**	(μg g^{-1})	19	**Ho**	(μg g^{-1})	4	**ΣHREE*[2]**	(μg g^{-1})	**157**
**MnO**	(wt.%)	0.03	**Nb**	(μg g^{-1})	106	**Mo**	(μg g^{-1})	17	**Eu**	(μg g^{-1})	4	**ΣREE*[3]**	(μg g^{-1})	**976**

*[1] ΣLREE group includes the lanthanide elements from La through Gd[8,9].

*[2] ΣHREE group includes the lanthanide elements from Tb through Lu, including Y[8,9].

*[3] ΣREE group comprises of the 15 lanthanide elements including Sc, and Y[8,9].

Supplementary Table S4: Natural radionuclides (Bq kg^{-1}) and total dose rate (nGy h^{-1}) of the studied bauxite residue / BR (red mud) and representative Greek bauxites (*n*=10)[10] compared with relevant values from literature[2,11-25].

GREEK BAUXITES: Radioactivity (Bq kg^{-1}) & Total Dose Rate (nGy h^{-1})							
**Greek bauxites (present study)**		**^{232}Th (due to ^{228}Ac)**	**^{238}U (due to ^{234}Th)**	**^{226}Ra**	**^{40}K**	**^{137}Cs**	**Total Dose Rate**
**Sample Code**	**Sample Description**						
ALM0306_PL1_B1	*Low grade; Fe-rich; Diasporic; Red-brown*	192	69	138	15	0	174
ALM0306_PL1_B3	*Low grade; Fe-rich; Diasporic; Red-brown*	184	89	130	57	0	177
ELM0206_DV_B1	*Low grade; Fe-rich; Diasporic; Red-brown*	179	44	115	15	0	154
SAB0306_ASV	*Low grade; Fe-rich; Diasporic; Red-brown*	203	42	96	16	0	162
ALM0306_PL1_B2	*Low grade; Fe-rich; Boehmitic; Red-brown*	159	54	129	60	0	151
ELM0206_KV_B1	*Low grade; Fe-rich; Boehmitic; Red-brown*	155	27	64	109	0	130
ELM0206_2H1	*Low grade; Fe-rich; Boehmitic; Red-brown*	161	60	145	108	0	150
SAB0306_SKR	*Low grade; Fe-rich; Boehmitic; Red-brown*	166	38	83	49	0	137
ALM0306_PL1_BIW	*High grade; Fe-depleted; Diasporic; White-grey*	229	121	144	17	0	200
ALM0306_PL1_BS2	*High grade; Fe-depleted; Diasporic; White-grey*	191	118	160	12	0	186
**Min – Max Values**		**155 – 229**	**27 – 121**	**64 – 160**	**17 – 117**	**0**	**130 – 200**
**Average Value**		**182**	**66**	**120**	**46**	**0**	**162**
**Greek bauxites (previous works)**							
Greek bauxites[11]		205 – 226	143 – 248	74 – 150	28		

Supplementary Table S4: (continued)

Bauxite Residue / BR (Red Mud) Radioactivity (Bq kg^{-1}) & Total Dose Rate (nGy h^{-1})						
**Greece**						
**Red Mud - Greece (present study)**	**355**	**133**	**171**	**45**	**0**	**295**
Red Mud - Greece[11]	15 – 412	52 – 400	13 – 185	72 – 160	1 – 5	
Red Mud - Greece[2]	472	149	379	21		
Red Mud - Greece[12]	346	182	232	45		
**Worldwide**						
Red Mud - Turkey[13]	539	218	210	112		
Red Mud - Turkey[14]	342 – 357		128 – 285	94 – 110		
Red Mud - Hungary[15]	219 – 392		225 – 568	5 – 101		
Red Mud - Hungary[16]	285 – 380		150 – 700	5 – 101		
Red Mud - Hungary[16]	87 – 545		102 – 506	47 – 212		
Red Mud - Hungary[17]	640	550		250	5.5	
Red Mud - Spain[18]	598	350	203	62		
Red Mud - Germany[19]	183	85	122			
Red Mud - Jamaica[20]	328 – 350		370 – 1047	265 – 335		
Red Mud - Australia[21]	1129		326	30		
Red Mud - Australia[22]		400	310	350		
Red Mud - Australia[23 and refs therein]	1000 – 1900	150 – 600		70 – 230		
Red Mud - China[24]	705	477		153		
Red Mud - China[25]	360 – 475		125 – 620	67 – 247		

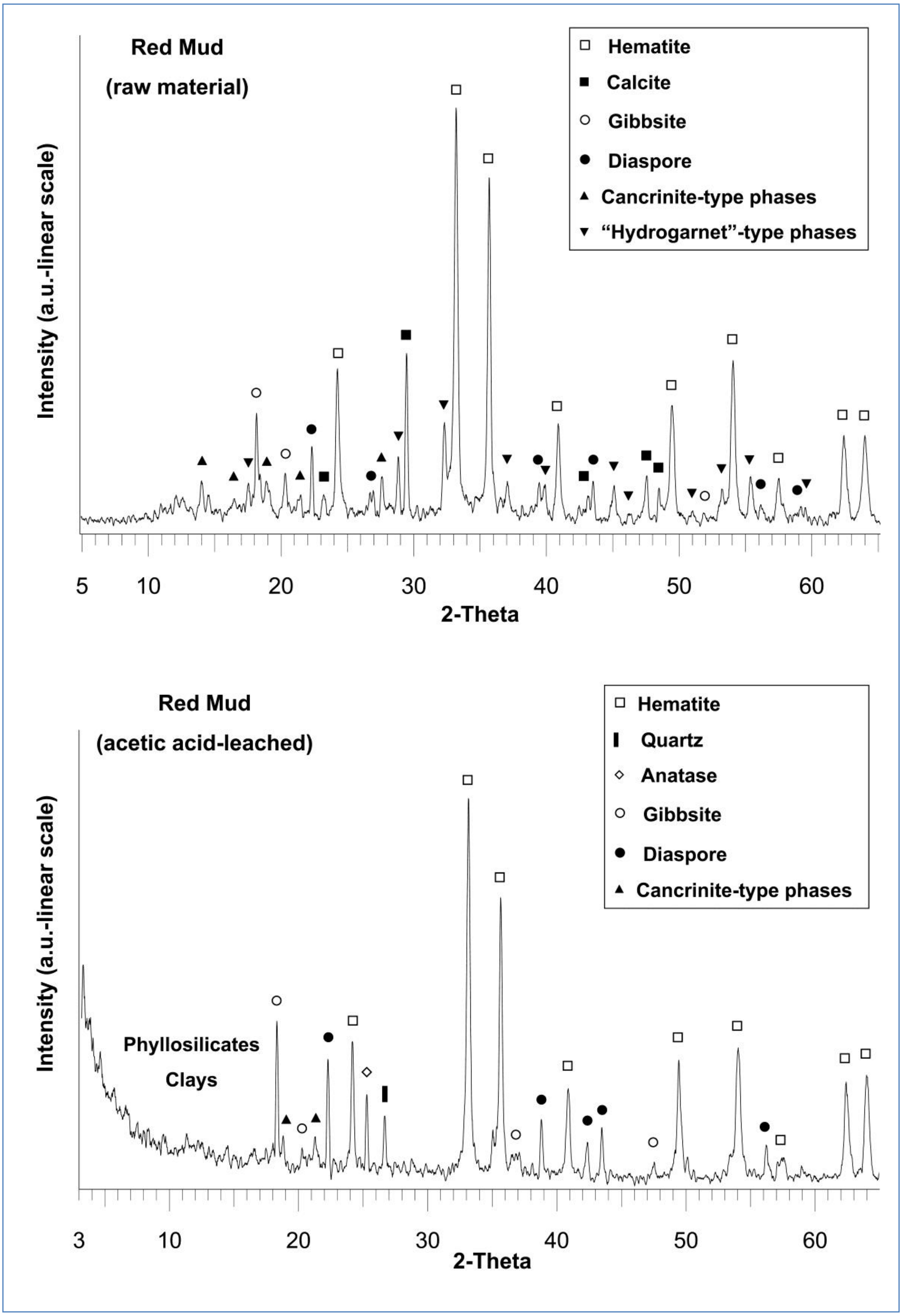

Supplementary Figure 1 | PXRD pattern of the studied bauxite residue / BR (red mud) from Greece (raw material: upper image) and the acetic acid-leached sample (lower image).

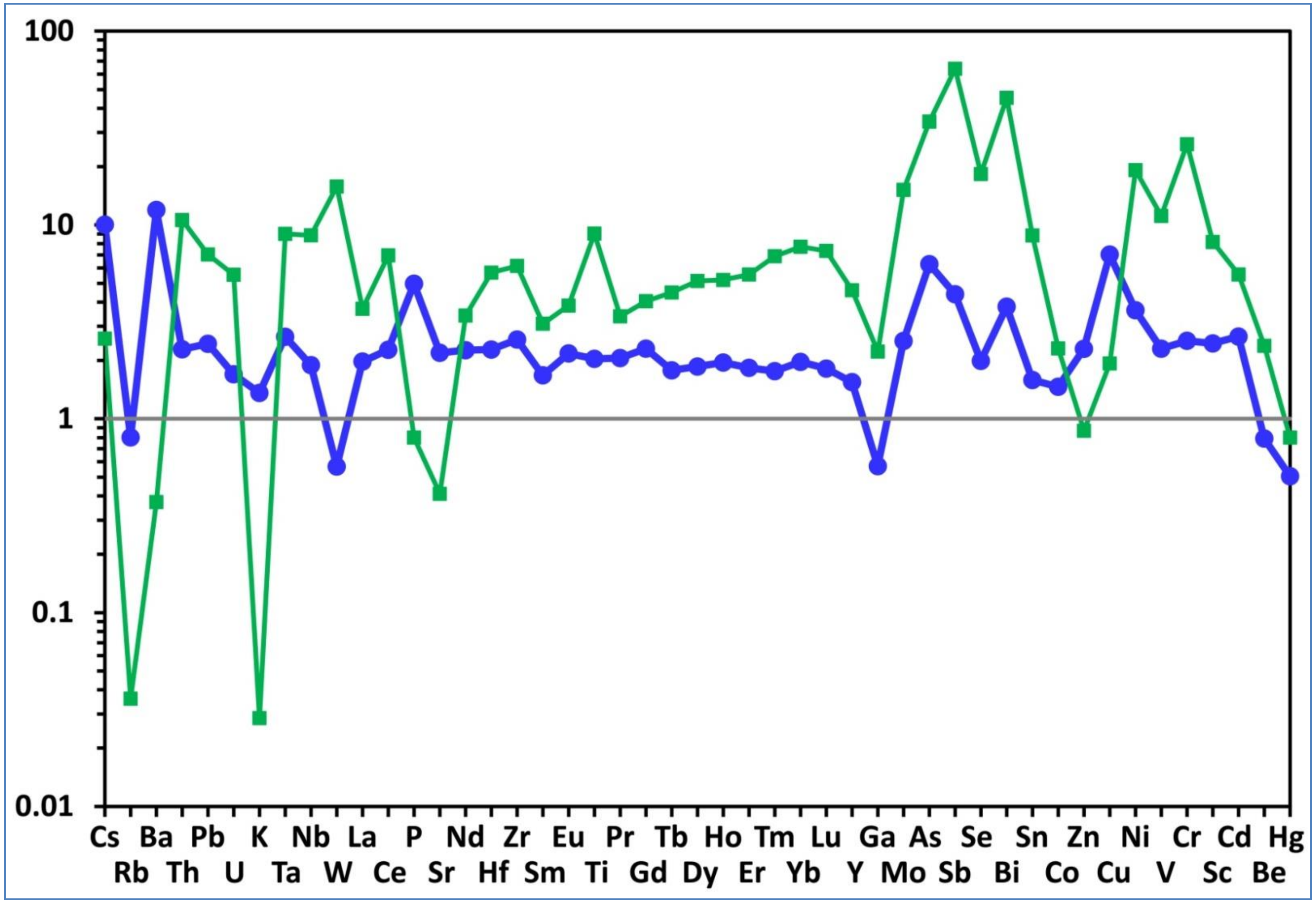

Supplementary Figure 2 | Spider diagrams of the studied bauxite residue / BR elemental content normalized: (a) to the average (n=16)[10] of the chemical composition of Greek bauxites from the Parnassos-Ghiona area, solid blue circles; (b) to the reference values of Upper Continental Crust/UCC[26], solid green squares.

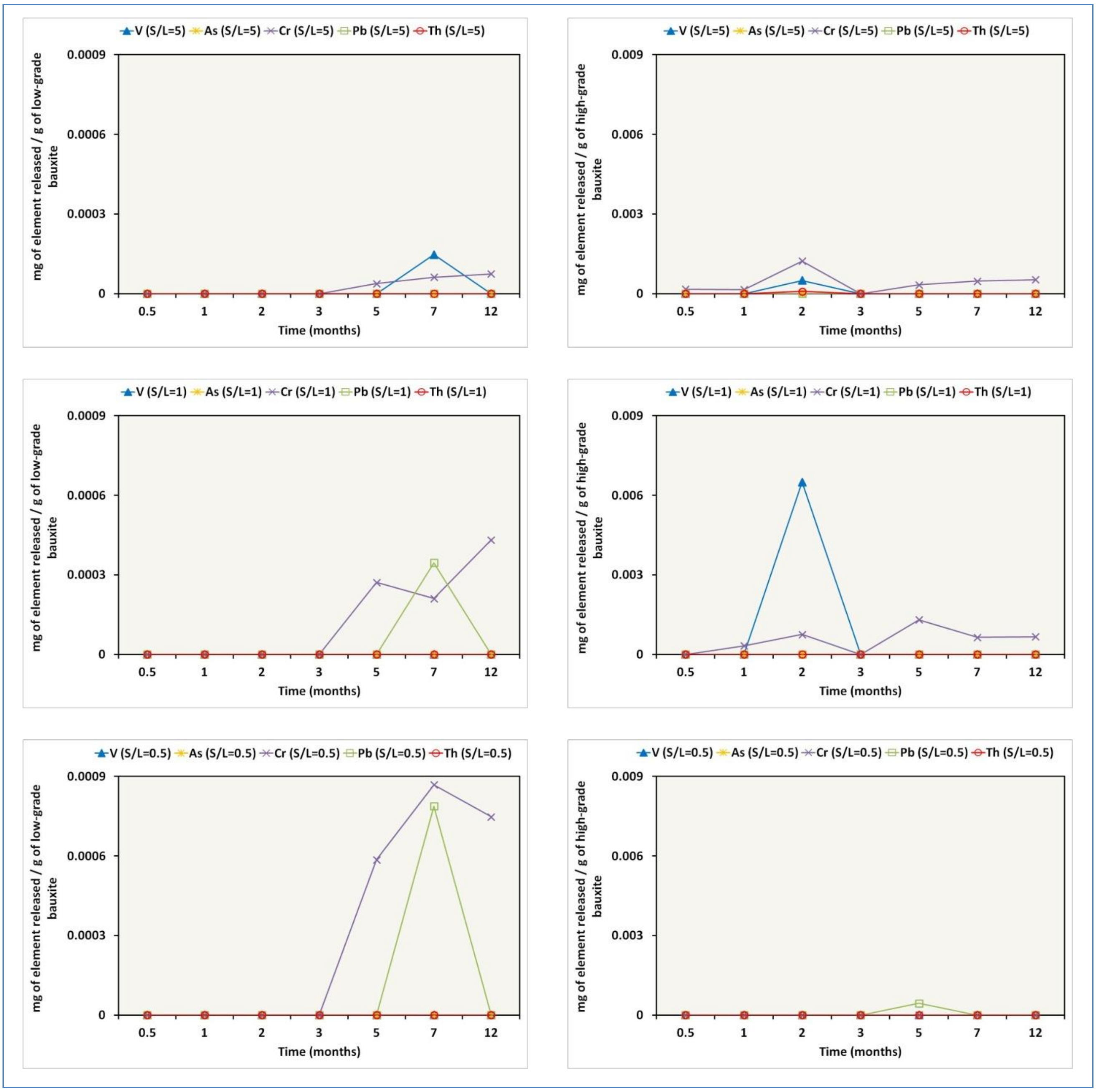

Supplementary Figure 3 | Results from leaching experiments showing the V, As, Cr, Pb, and Th negligible release from typical low-grade (left images) and high-grade (right images) Greek bauxite leached by Mediterranean seawater from Greece.

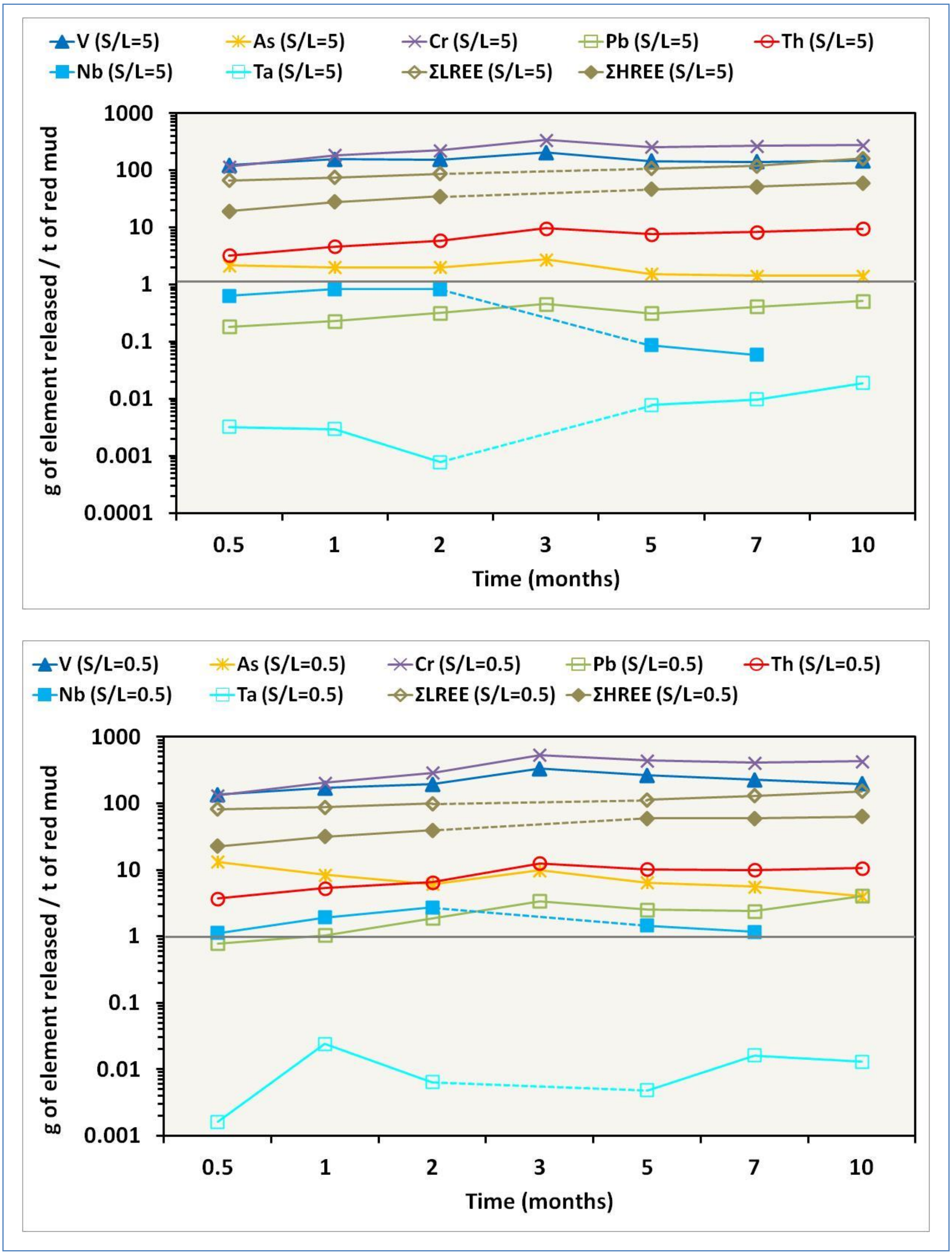

Supplementary Figure 4 | Results from leaching experiments showing the release of ΣLREE, and ΣHREE, along with with Cr, V, Th, As, Pb, Nb, and Ta from Greek bauxite residue / BR (red mud) leached by concentrated acetic acid.

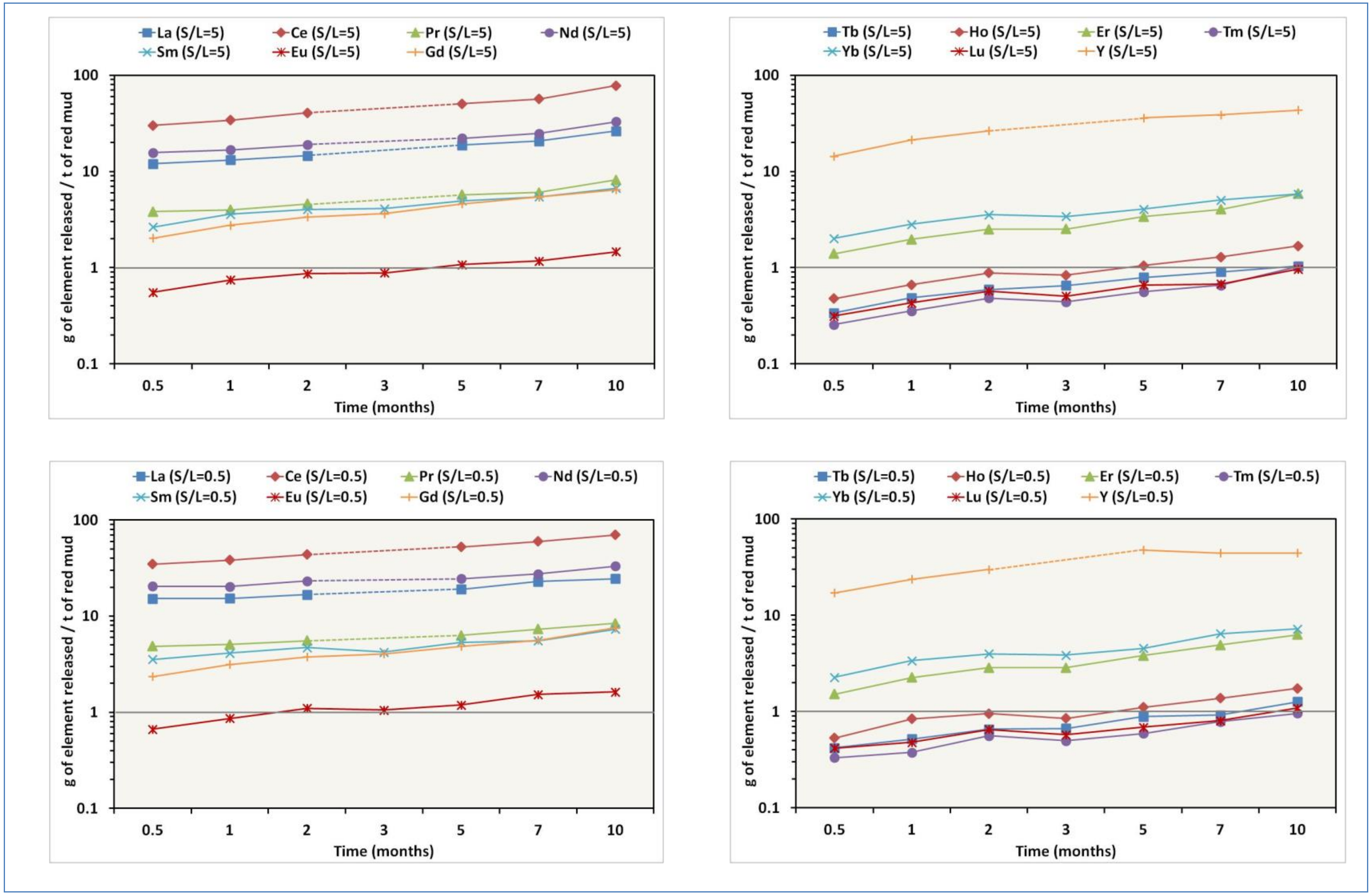

Supplementary Figure 5 | Results from leaching experiments showing the LREE (La, Ce, Pr, Nd, Sm, Eu, and Gd; left images) as well as the HREE (Tr, Ho, Er, Tm, Yb, Lu, and Y; right images) release (g) per 1 ton of Greek bauxite residue / BR (red mud) leached by concentrated acetic acid.

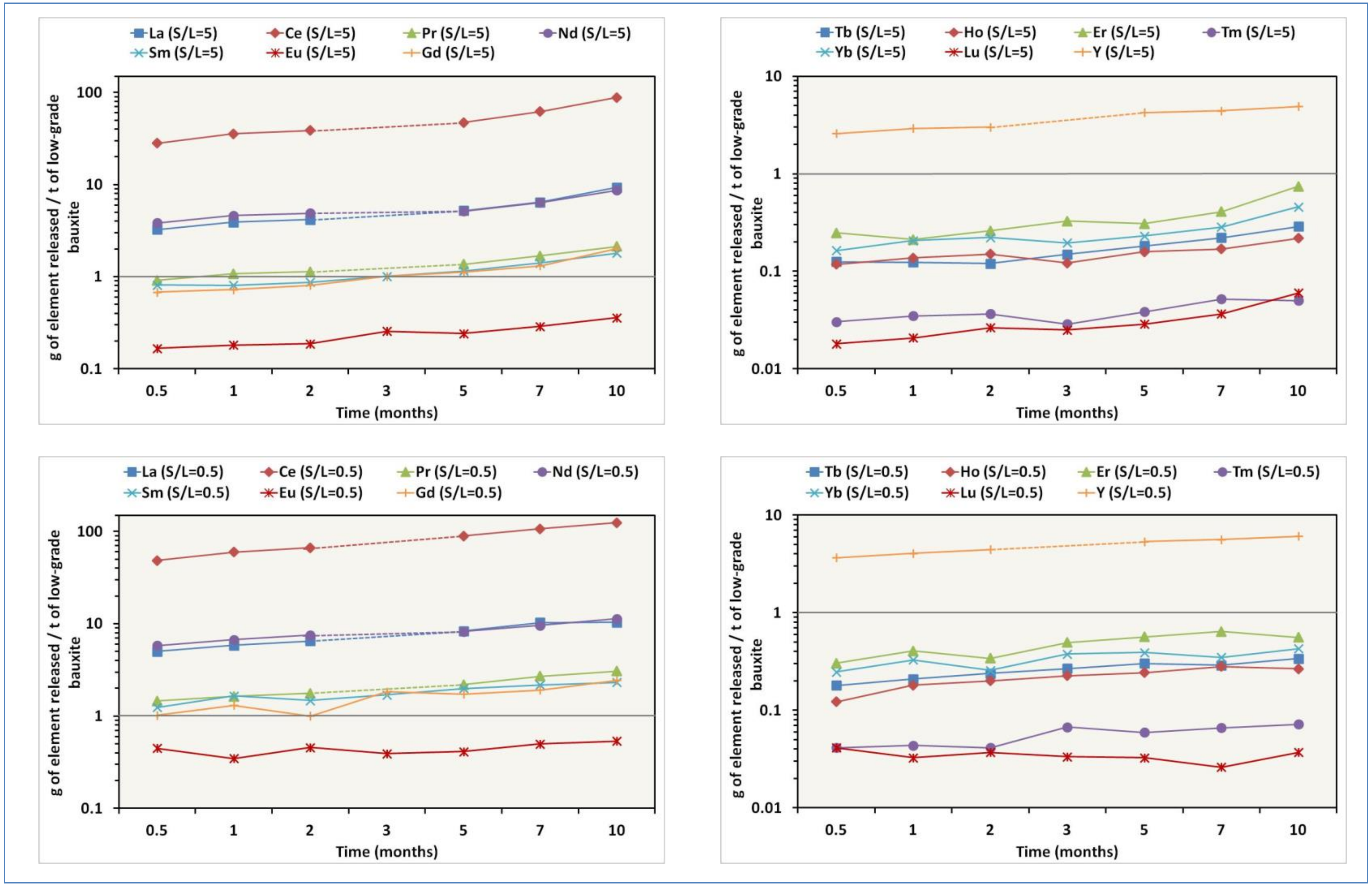

Supplementary Figure 6 | Results from leaching experiments, relative to S/L ratio effect, showing the LREE (La, Ce, Pr, Nd, Sm, Eu, and Gd; left images) as well as the HREE (Tr, Ho, Er, Tm, Yb, Lu, and Y; right images) release from Greek typical low-grade bauxites leached by concentrated acetic acid.

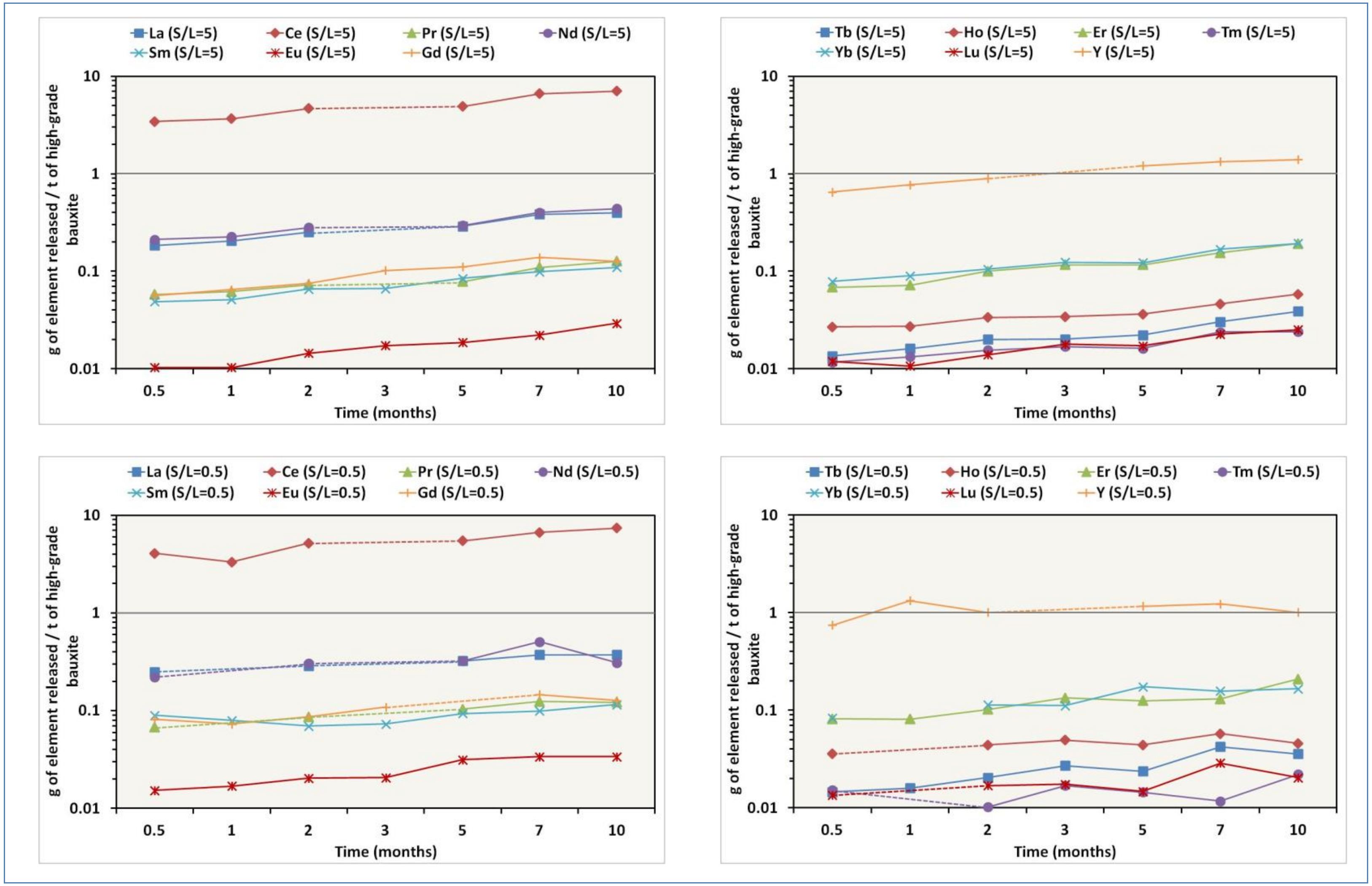

Supplementary Figure 7 | Results from leaching experiments, relative to S/L ratio effect, showing the LREE (La, Ce, Pr, Nd, Sm, Eu, and Gd; left images) as well as the HREE (Tr, Ho, Er, Tm, Yb, Lu, and Y; right images) release from Greek high-grade bauxites leached by concentrated acetic acid.

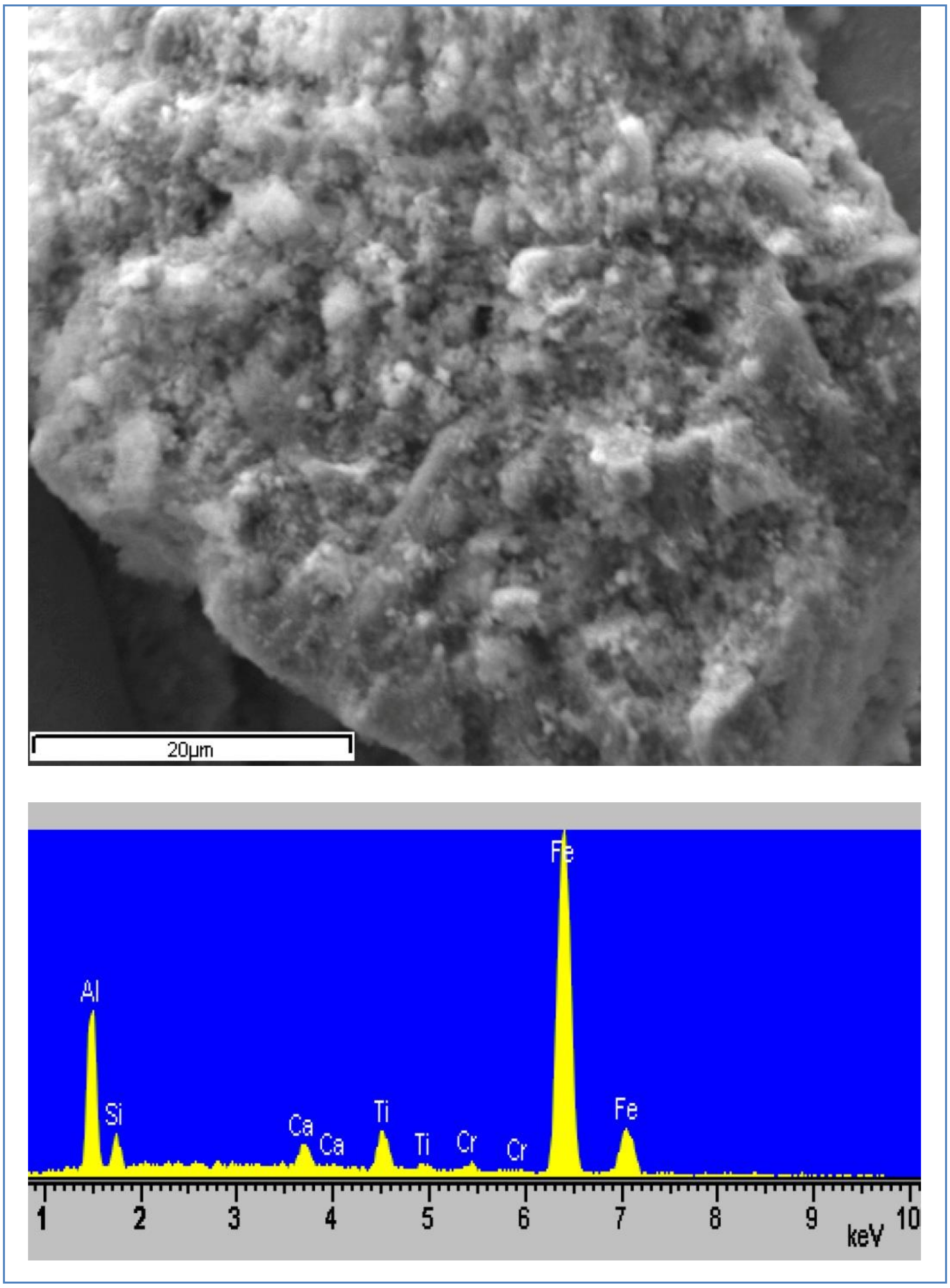

Supplementary Figure 8 | Morphology and chemical composition (major elements) of the studied bauxite residue / BR (red mud) by SEM-EDS at the microscale, after acid-leaching.

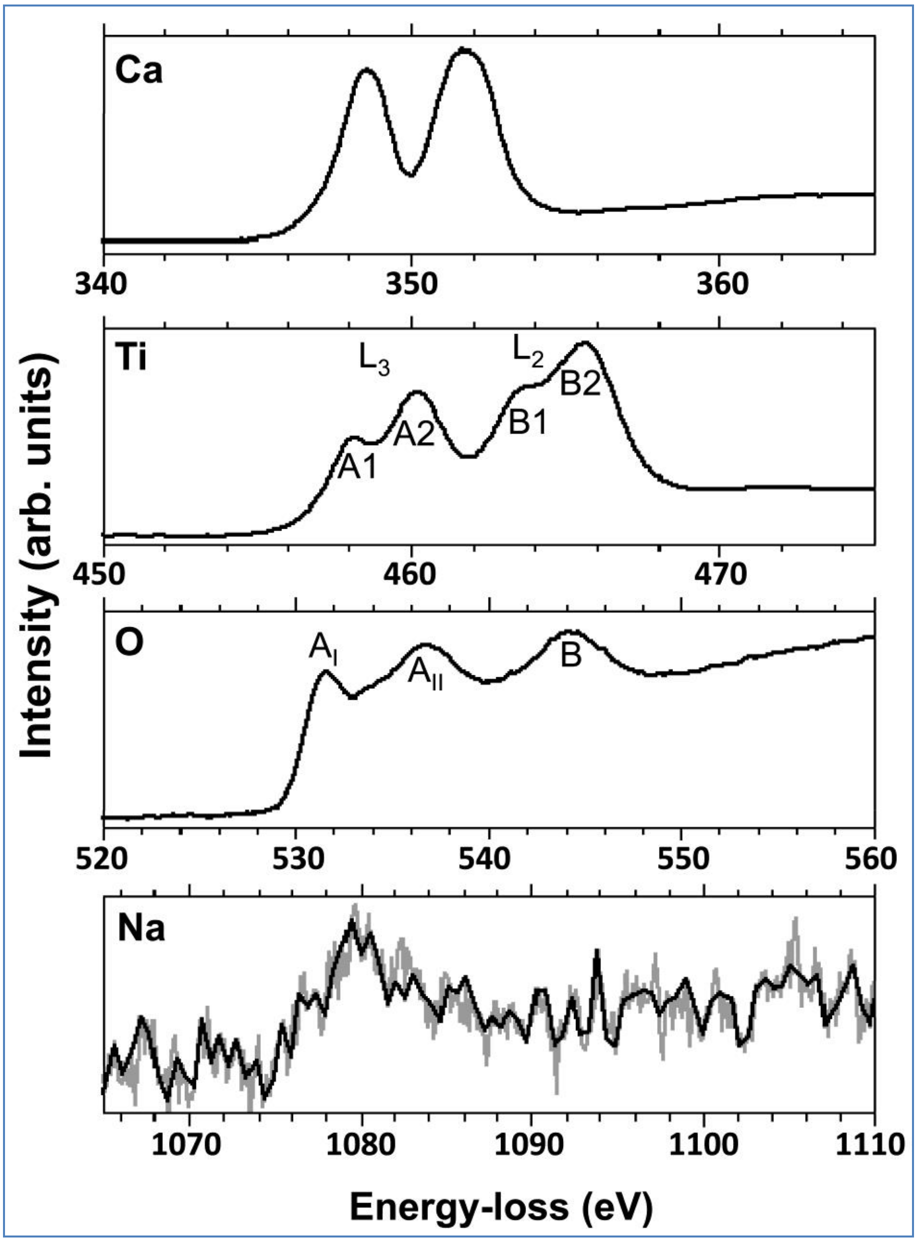

Supplementary Figure 9 | Representative Ca $L_{2,3}$-, Ti $L_{2,3}$-, O *K*- and Na *K*-edge EEL spectral data of the nano-perovskite in the Greek bauxite residue / BR (red mud).

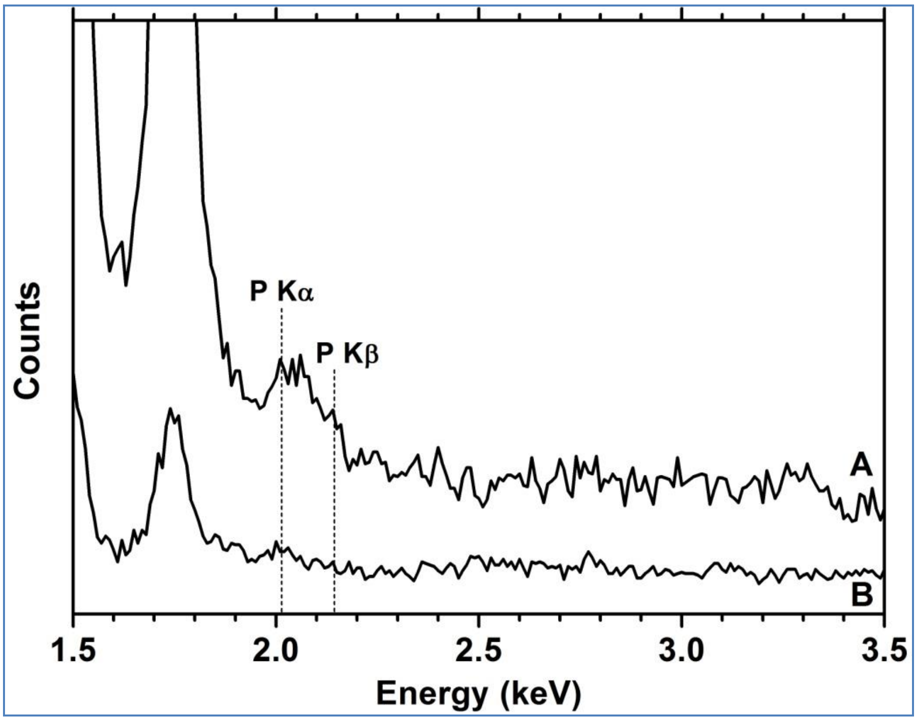

Supplementary Figure 10 │ The appearance of the P Kα peak (2.01 KeV) at the STEM-EDS spectrum of a Ti-containing hematite particle (**A**: hematite [211]; see also **Fig. 4**), in contrast to the absence of the P Kα peak at the STEM-EDS spectrum of another Ti-containing hematite particle (**B**: hematite [241]). Both the Ti-containing hematite particles observed in the "Al-Fe-Ca-Ti-Si-Na-Cr matrix" of the studied Greek bauxite residue / BR (red mud). A small contribution from the P Kβ peak (2.14 KeV) at the Ti-containing hematite [211] cannot be excluded.

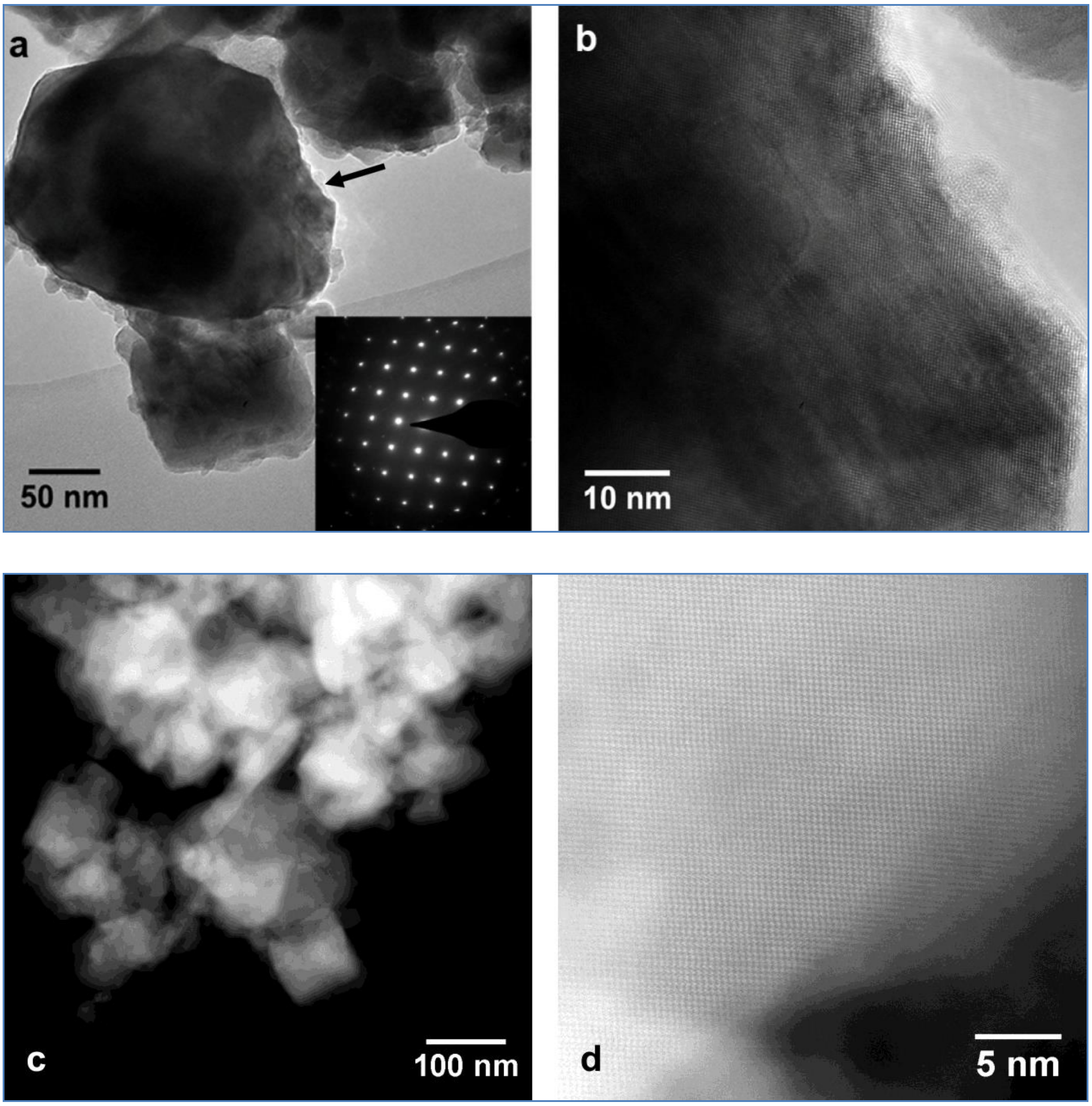

Supplementary Figure 11 | *Upper images*: HRTEM data, including a BF image and its SAED pattern (**a**), of a representative Ti-oxide particle (anatase / TiO_2), which exists in the "Al-Fe-Ca-Ti-Si-Na-Cr matrix" of the studied Greek bauxite residue / BR (red mud). The viewing direction for its SAED pattern is [111]. Several planar defects, common in anatase, can be observed in the HRTEM image (**b**). *Lower images*: STEM-HAADF images of the anatase, showing no apparent sign of Th. The HAADF contrast is proportional to the square of a mean atomic number.

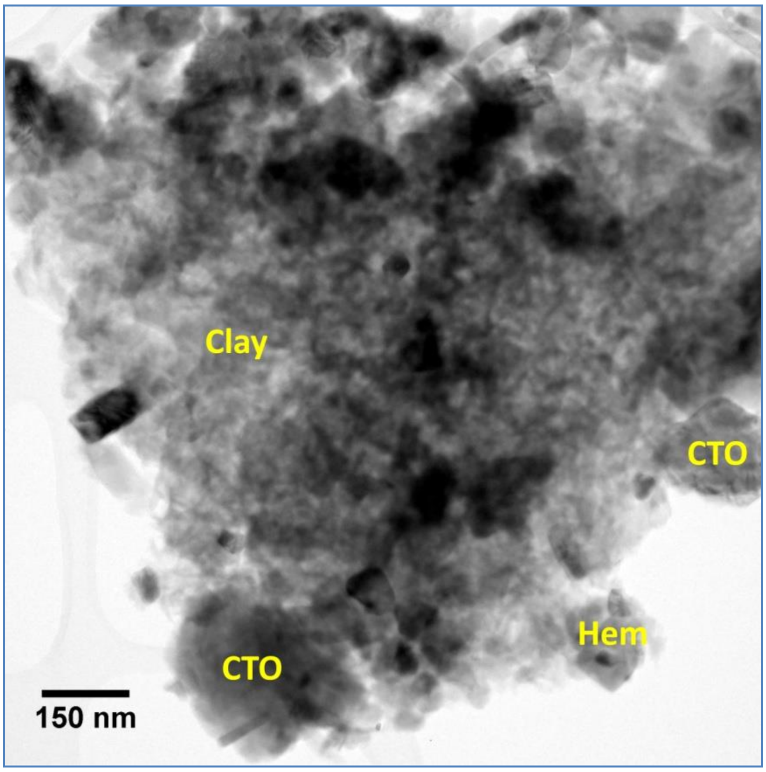

Supplementary Figure 12 | BF image of the Th-free clay-like phases together with nano-perovskite (CTO) and Th-free Ti-containing hematite (Hem) co-existing into the "Al-Fe-Ca-Ti-Si-Na-Cr matrix" of the studied Greek bauxite residue / BR (red mud).

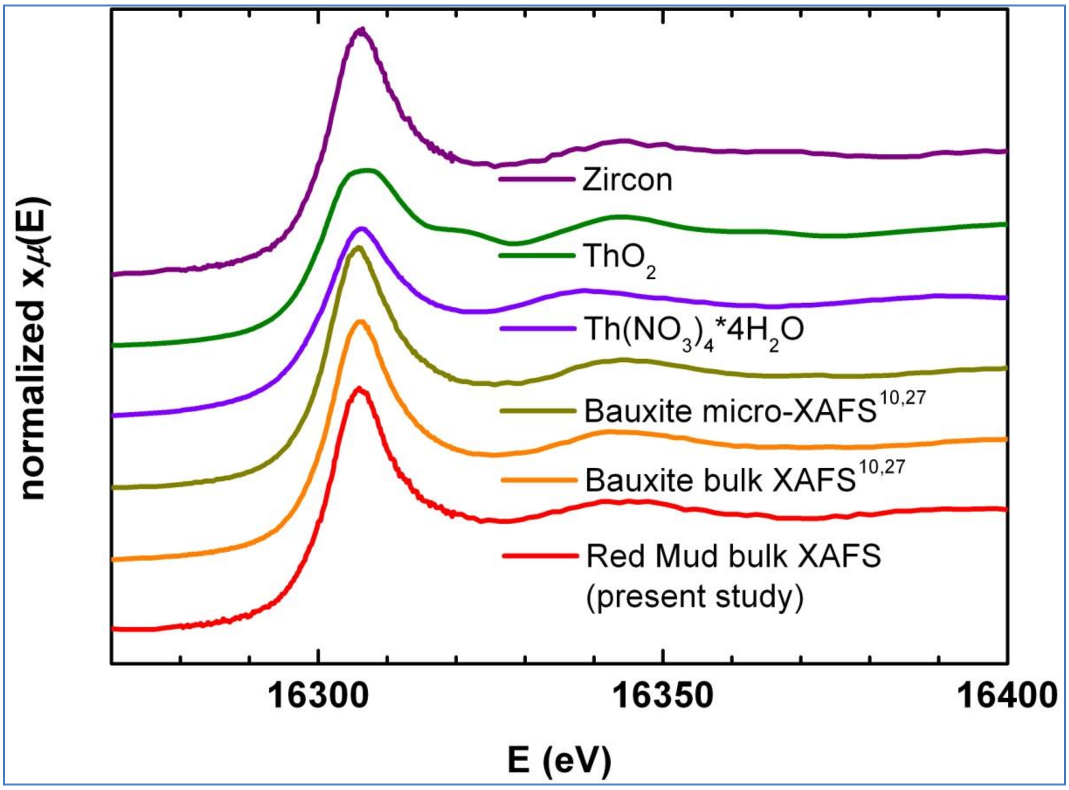

Supplementary Figure 13 | Th L_{III}-edge bulk XANES of the studied Greek bauxite residue / BR (red mud; present study) together with bulk & micro-XANES of Greek bauxite[10,27], compared with spectra of reference materials.

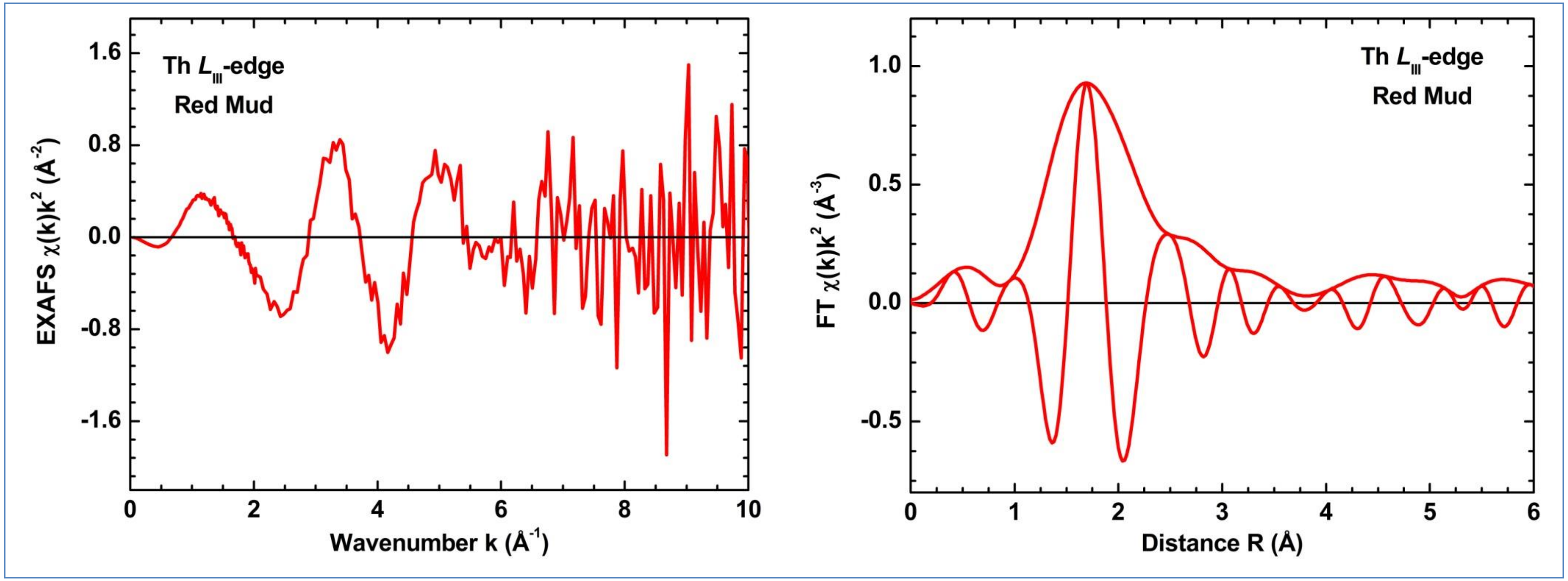

Supplementary Figure 14 | Experimental Th L_{III}-edge EXAFS spectrum (left image) and its Fourier transform – FT (right image) of the studied bauxite residue / BR (red mud).

Supplementary References

1. Grew, E. S. *et al*. Nomenclature of the garnet supergroup. *Am. Mineral*. **98,** 785-811 (2013).

2. Pontikes, Y., Vangelatos, I., Boufounos, D., Fafoutis, D. & Angelopoulos, G. N. Environmental aspects on the use of Bayer's process bauxite residue in the production of ceramics. Paper presented at: The 11th International Ceramics Congress, Sicily, Italy. Switzerland: Trans Tech Publications. (DOI:10.4028/www.scientific.net/AST.45.2176) (2006, June 4-9)

3. Burke, I. T. *et al*. Speciation of arsenic, chromium, and vanadium in red mud samples from the Ajka spill site, Hungary. *Environ. Sci. Technol*. **46,** 3085-3092 (2012).

4. Liang, W. *et al*. Effect of strong acids on red mud structural and fluoride adsorption properties. *J. Colloid Interf. Sci.* **423,** 158-165 (2014).

5. Smičiklas, I. *et al*. Effect of acid treatment on red mud properties with implications on Ni(II) sorption and stability. *Chem. Engineer. J.* **242,** 27-35 (2014).

6. Calvert, C. C., Rainforth, W. M., Sinclair, D. C. & West, A. R. EELS characterization of bulk $CaCu_3Ti_4O_{12}$ ceramics. *Micron.* **37,** 412-419 (2006).

7. Mariano, A. N. & Mariano, A. Jr. Rare earth mining and exploration in North America. *Elements*. **8,** 369-376 (2012).

8. Gambogi, J. 2012 *Minerals Yearbook: Rare Earths [Advance Release]*. U.S. Department of the Interior, USGS, Technical Report. (2015) Available at: http://minerals.usgs.gov/minerals/pubs/commodity/rare_earths/myb1-2012-raree.pdf. (Accessed: 24th February 2015)

9. Zepf, V. (2013) Rare Earth Elements: What and where they are. In *Rare Earth Elements: A new approach to the nexus of supply, demand, and use: Exemplified along the use of Neodymium in permanent magnets*; Zepf, V., Ed.; Springer Theses; Springer-Verlag Berlin Heidelberg, 2013; pp 11-39.

10. Gamaletsos, P. Mineralogy and geochemistry of bauxites from Parnassos-Ghiona mines and the impact on the origin of the deposits. Ph.D. Thesis, University of Athens, Greece, 2014.

11. Papatheodorou, G., Papaefthymiou, H., Maratou, A. & Ferentinos, G. Natural radionuclides in bauxitic tailings (red-mud) in the Gulf of Corinth, Greece. *Radioprotection*. **40,** 549-555 (2005).

12. Samouhos, M., Taxiarchou, M., Tsakiridis, P. E. & Potiriadis, K. Greek "red mud" residue: A study of microwave reductive roasting followed by magnetic separation for a metallic iron recovery process. *J. Hazard. Mater.* **254-255,** 193-205 (2013).

13. Akinci, A. & Artir, R. Characterization of trace elements and radionuclides and their risk assessment in red mud. *Mater. Charact.* **59,** 417-421 (2008).

14. Turhan, Ş., Arıkan, İ. H., Demirel, H. & Güngör, N. Radiometric analysis of raw materials and end products in the Turkish ceramics industry. *Radiat. Phys. Chem*. **80,** 620-625 (2011).

15. Somlai, J., Jobbágy, V., Kovács, J., Tarján, J. & Kovács, T. Radiological aspects of the usability of red mud as building material additive. *J. Hazard. Mater.* **150,** 541-545 (2008).

16. Jobbágy, V., Somlai, J., Kovács, J., Szeiler, G. & Kovács, T. Dependence of radon emanation of red mud bauxite processing wastes on heat treatment. *J. Hazard. Mater.* **172,** 1258-1263 (2009).

17. Ruyters, S. *et al*. The red mud accident in Ajka (Hungary): plant toxicity and trace metal bioavailability in red mud contaminated soil. *Environ. Sci. Technol*. **45,** 1616-1622 (2011).

18. Rubinos, D. A. & Barral, M. T. Fractionation and mobility of metals in bauxite red mud. *Environ. Sci. Pollut. Res.* **20,** 7787-7802 (2013).

19. Von Philipsborn, H. & Kühnast, E. Gamma spectrometric characterization of industrially used African and Australian bauxites and their red mud tailings. *Radiat. Prot. Dosim.* **45,** 741-743 (1992).

20. Pinnock, W. R. Measurements of radioactivity in Jamaican building materials and gamma dose equivalents in a prototype red mud house. *Health Phys.* **61,** 647-651 (1991).

21. Beretka, J. & Mathew, P. J. Natural radioactivity of Australian building materials, industrial wastes and by-products. *Health Phys.* **48,** 87-95 (1985).

22. Cooper, M. B., Clarke, P. C., Robertson, W., McPharlin, I. R. & Jeffrey, R. C. An investigation of radionuclide uptake into food crops grown in soils treated with bauxite mining residues. *J. Radioanal. Nucl. Chem.* **194,** 379-387 (1995).

23. Cooper, M. B. *Naturally Occurring Radioactive Materials (NORM) in Australian Industries - Review of Current Inventories and Future Generation*, Radiation Health and Safety Advisory Council,

Technical Report. (2005) Available at: http://www.arpansa.gov.au/pubs/norm/cooper_norm.pdf. (Accessed: 21st December 2005)

24. Wang, K. Levels of radioactivity in the red mud and red mud cement and its dose rate for local residents. *Huanjing Kexue*. **13,** 90-93 (1992).

25. Wang, P. & Liu, D. -Y. Physical and chemical properties of sintering red mud and Bayer red mud and the implications for beneficial utilization. *Materials*. **5,** 1800-1810 (2012).

26. Rudnick, R. & Gao, S. Composition of the continental crust in *Treatise on Geochemistry* , Vol. 3 (eds Holland, H. D. & Turekian, K. K.) 1-64 (Elsevier, 2003).

27. Gamaletsos, P. *et al*. Thorium partitioning in Greek industrial bauxite investigated by synchrotron radiation and laser-ablation techniques. *Nucl. Instrum. Meth. B.* **269,** 3067-3073 (2011).
